# Comprehensive analysis of atherosclerotic plaques reveals crucial genes and molecular mechanisms associated with plaque progression and rupture

**DOI:** 10.3389/fcvm.2023.951242

**Published:** 2023-03-28

**Authors:** Guoqi Zhu, Yanhua Gao, Jun Qian, Yan Lai, Hao Lin, Chengxing Liu, Fei Chen, Xuebo Liu

**Affiliations:** Department of Cardiology, Tongji Hospital, Tongji University School of Medicine, Shanghai, China

**Keywords:** WGCNA, DEGs, plaque rupture, miRNAs, biomarker, acute myocardial infarction

## Abstract

**Background:**

Plaque rupture and acute atherothrombosis, resulting from continued progression of atherosclerotic plaques (APs), are major contributors to acute clinical events such as stroke or myocardial infarction. This article aimed to explore the gene signatures and potential molecular mechanisms in the progression and instability of APs and to identify novel biomarkers and interventional targets for AP rupture.

**Methods:**

The microarray data were downloaded from the Gene Expression Omnibus (GEO) database and grouped into discovery and validation cohorts. In the discovery cohort, Weighted Gene Co-Expression Network Analysis was performed for finding co-expression modules, and the Metascape database was used to perform functional enrichment analysis. Differential Expression Genes analysis subsequently was performed in the validation cohort for verification of the obtained results. Common genes were introduced into Metascape database for protein–protein interaction and functional enrichment analysis. We constructed the miRNAs–mRNAs network with the hub genes. Moreover, gene expression profiles of peripheral blood mononuclear cells (PBMCs) from peripheral blood of patients with plaque rupture were analyzed by high-throughput sequencing, and the diagnostic power of hub genes was verified by receiver operating characteristic (ROC) analysis.

**Results:**

In the discovery cohort, the brown module in GSE28829 and the turquoise module in GSE163154 were the most significant co-expression modules. Functional enrichment analysis of shared genes suggested that “Neutrophil degranulation” was the most significantly enriched pathway. These conclusions were also demonstrated by the validation cohort. A total of 16 hub genes were identified. The miRNA–mRNA network revealed that hsa-miR-665 and hsa-miR-512-3p might regulate the “Neutrophil degranulation” pathway through *PLAU* and *SIRPA*, which might play a significant role in AP progression and instability. Five hub genes, including *PLAUR*, *FCER1G*, *PLAU*, *ITGB2*, and *SLC2A5*, showed significantly increased expression in PBMCs from patients with plaque rupture compared with controls. ROC analysis finally identified three hub genes *PLAUR*, *FCER1G*, and *PLAU* that could effectively distinguish patients with APs rupture from controls.

**Conclusions:**

The present study demonstrated that the “neutrophil degranulation” signaling pathways and identified novel mRNA and miRNA candidates are closely associated with plaque progression and instability. The hub genes *FCER1G*, *PLAUR*, and *PLAU* may serve as biomarkers for the prospective prediction of AP rupture.

## Introduction

Atherosclerosis (AS) and atherosclerotic cardiovascular disease (CVD) is the leading cause of morbidity and mortality worldwide (1). AS is a chronic inflammatory disease mainly characterized by the deposition of lipids and fibrous material in the subintima of arterial vessels (2). Continued local accumulation of lipids and lipid-engorged cells leads to the formation and progression of atherosclerotic plaques (APs), which are composed of a protective fibrous cap and nidus of a lipid­rich or necrotic core (2, 3). Once established, APs progress continuously and become more fibrotic (4).

Not all APs are the same, with some plaques remaining stable or quiescent for years, whereas others progress continuously and become unstable and vulnerable (5–7). Advanced APs can invade the arterial lumen, hinder blood flow, lead to tissue ischemia, and are more likely to rupture and cause thromboembolism, which is the most common cause of myocardial infarction (8, 9). Due to the slow progression of AS, most cases have been asymptomatic for decades. When symptoms appear, they are often related to thrombotic obstruction caused by the rupture of APs and can cause severe consequences (9–11).

Intraplaque hemorrhage (IPH), caused by the rupture of the fibrous cap or leakage of blood vessels due to extensive neovascularization within atheromatous plaques, is another typical histological feature of plaque instability (12, 13). IPH is a common feature of high rupture risk coronary plaques and may also be a trigger for plaque instability. Virmani and Roberts found extravasated erythrocytes in 84% of coronary plaques from 57 patients who died of coronary artery disease (14). In another study, Kolodgie et al. found that IPH was present in 77% of high-risk thin-cap APs (15). Therefore, the development of a reliable tool to identify high-risk APs or predict their rupture risk is important for the clinical prevention of acute myocardial infarction (AMI).

In recent years, comprehensive histopathological and related high-throughput gene profiling studies on atherosclerotic plaque samples have greatly enhanced our understanding of the mechanisms of plaque pathogenesis and progression (16–18). By contrasting genomic expression differences in different arterial plaque tissues, researchers have identified several genes and pathways that are involved in the progression of AS and APs (17–20).

In this study, we integrated gene expression data from the Gene Expression Omnibus (GEO) database on advanced plaques, high-risk plaques, and ruptured plaques and divided them into discovery and validation groups. Two powerful analytical approaches, Differential Expression Genes (DEGs) analysis and Weighted Gene Co-Expression Network Analysis (WGCNA), were employed to define high rupture risk plaque-specific genes (21, 22). We further detected the expression patterns of these genes in the blood of patients with plaque rupture to observe whether these genes can be used as molecular markers of potentially high-risk plaques and improve diagnostics for underlying arterial plaque fissure and rupture. That may shed new light on the prevention and treatment of AS and may offer new therapeutic targets for AS or new biomarkers for AMI diagnosis.

## Materials and method

### Microarray datasets

**Figure 1:**
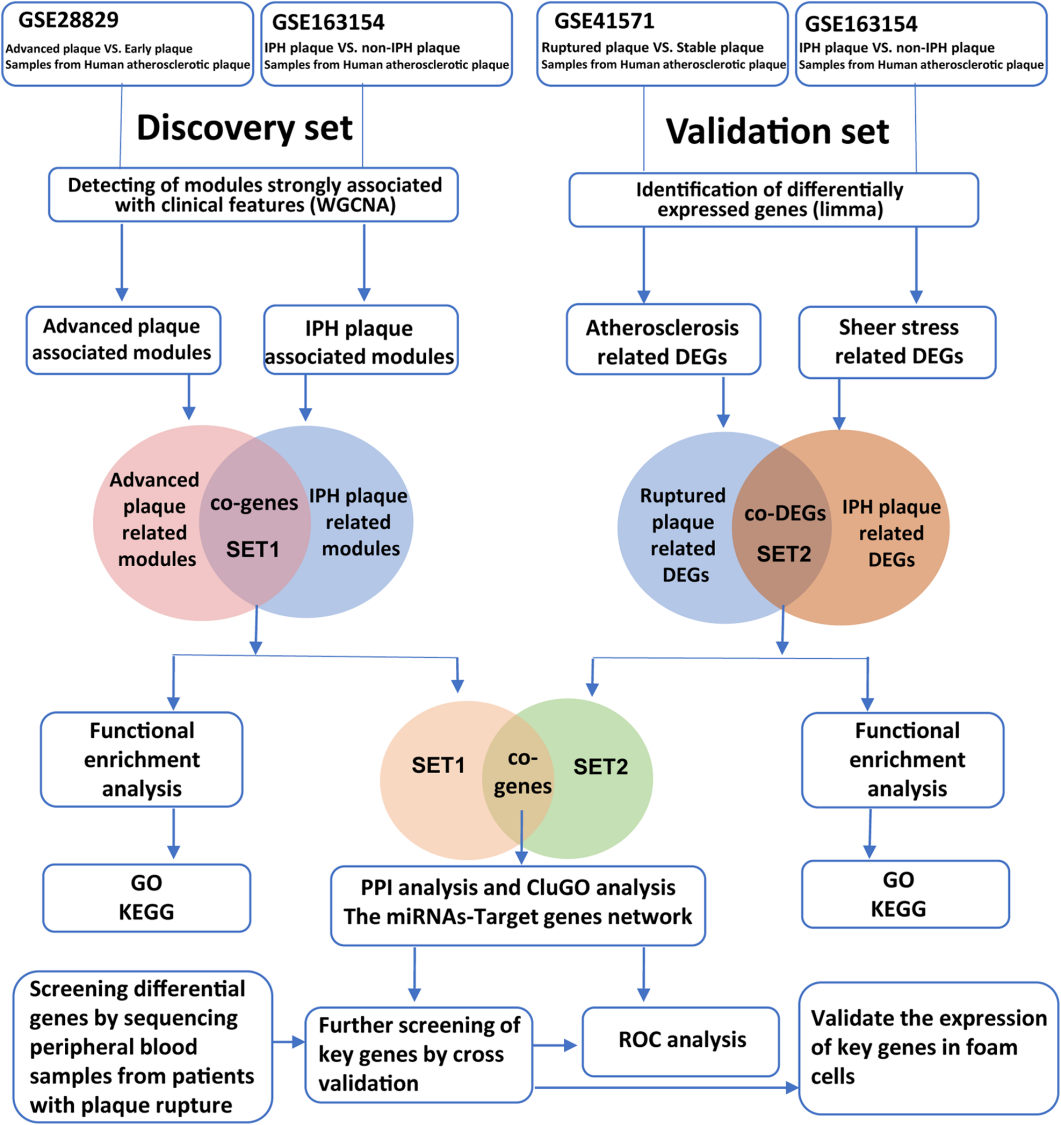
Workflow of bioinformatics analysis.

In order to identify key genes closely related to APs’ progression and rupture, we screened gene expression profiles related to advanced plaque, high rupture risk plaque, or ruptured plaque in the GEO database (https://www.ncbi.nlm.nih.gov/geo). Finally, the raw gene expression profiles GSE163154, GSE28829, and GSE41571 were downloaded from the GEO database for the next analysis. The GSE28829 expression profile dataset consists of 13 early and 16 advanced human carotid plaque samples (23), while GSE163154 involved 16 low-risk (absence of IPH) and 27 high-risk (presence of IPH) atherosclerotic lesion segments from carotid endarterectomy patients (17). The GSE41571 dataset consists of five ruptured and six stable plaques (24). In addition, to validate the expression patterns of hub genes in the blood of AMI patients, we downloaded the dataset GSE66360 from the GEO database, which contained plasma samples from 49 AMI patients and 50 healthy controls (25). A schematic diagram of our workflow is shown in Figure 1.

### Construction of a co-expression network with WGCNA

WGCNA is an efficient method for detecting gene co-expression networks and gene modules that are highly correlated with disease (21). The “WGCNA” package in R was used to construct WGCNA gene co-expression networks and modules by using the standard procedure. The “WGCNA” package was employed to obtain gene modules that have a close relationship with high-risk (presence of IPH) plaque samples in GSE163154. In GSE28829, we mostly focus on modules that are closely related to advanced plaques. To improve the sensitivity and accuracy, we selected the top 25% genes with highest variation based on variance. Cluster analysis was performed using the “Hclust” function in R studio software, which uses the complete linkage method for hierarchical clustering, to exclude outlier samples. Appropriate soft threshold power values were calculated using the “pickSoftThreshold” function in the “WGCNA” package. This soft threshold power value is substituted into the formula amn = |cmn|*β* (amn denotes adjacency matrix between gene m and gene N, CMN denotes Pearson correlation coefficient between gene m and gene N, and *β* denotes the soft power value) to create a weighted adjacency matrix and further calculate gene modules closely related to clinical features. Selecting modules associated with advanced plaques and high-risk plaques for the next analysis.

### Functional and pathway enrichment analysis

Modules highly relevant to advanced plaques in GSE28829 were intersected with modules associated with high-risk plaques in GSE163154, and the obtained overlapped genes were called “Gene Set-1” (SET-1). The Metascape database is a web-based portal that combines functional enrichment, interactome analysis, gene annotation, and membership search to leverage over 40 independent knowledge bases to provide experimental biologists with a comprehensive resource of gene list annotation and analysis (26). Functional and pathway enrichment of SET-1 was performed with the Metascape database to explore the potential roles of these genes in plaque progression and plaque rupture.

### DEGs analysis in discovery cohorts

We performed the DEGs analysis on GSE163154 and GSE41571 to extract DEGs between high-risk plaque samples (ruptured plaque in GSE41571) and stable plaque samples. Uniform manifold approximation and projection (UMAP) analysis was used to detect the discrimination between different types of samples and was performed using the GEO2R online tool (27). DEGs analysis was performed the with limma package with |log FC| > 1 and *P-*value < 0.05 (22). We integrated the DEGs from GSE163154 and GSE41571 and obtained a single set of common genes, called “Gene Set-2” (SET-2). We performed functional enrichment analysis of SET-2 using the same method to assess the reliability of our analysis results obtained in the discovery cohorts.

### Identification of hub genes

To finally identify reliable hub genes, we focused on the intersection of SET-1 and SET-2. We retained the intersection of the two lists of genes and imported them into the Metascape database for protein–protein interaction (PPI) network and cluster analysis. In Metascape, PPI enrichment analysis has been carried out with the STRING and BioGrid databases, and the Molecular Complex Detection (MCODE) algorithm has been applied to identify densely connected network components (28–30).

To further validate the potential roles of hub genes in AP progression and plaque rupture, functional enrichment analysis of hub genes was performed using the ClueGO plug-in in Cytoscape software and visualize the GO terms as functionally grouped networks (31).

### Construction of miRNAs–target genes network

MicroRNAs (miRNAs) are small noncoding RNAs that can regulate gene expression by inhibiting mRNA translation or promoting mRNA degradation and can also unconventionally activate gene transcription by targeting enhancers (32, 33). We further explored whether some miRNAs are involved in the regulation of these hub genes. The miRTarBase is an annotated comprehensive, experimentally validated miRNA target interaction database in miRNA-related research fields, which contains 4,076 miRNAs and 23,054 target genes from more than 8,500 articles (34). ENCORI is an open-source platform for studying miRNA target interactions, containing more than 2.9 million miRNA–mRNA interactions identified from multidimensional sequencing data (35). The overlapped miRNAs of hub genes in miRTarBase and ENCORI were subsequently applied for constructing the miRNAs–mRNAs regulated network in Cytoscape software.

### Identification of patients with plaque rupture

Twenty-nine patients who underwent coronary angiography (CAG) between March and October 2021 in Shanghai Tongji Hospital were enrolled in this study, including 19 AMI patients with plaque rupture who underwent percutaneous coronary intervention (PCI) and 10 patients who underwent CAG but without myocardial infarction. All patients received a loading dose of aspirin 300 mg and a P2Y12 receptor inhibitor (180 mg ticagrelor) followed by 100 mg once daily dose of aspirin and 90 mg twice daily dose of ticagrelor. After completion of diagnostic CAG, the culprit lesion was determined on the basis of ECG, echocardiography, and CAG findings. Optical coherence tomography (OCT) examination was performed using a frequency-domain (C7-XR, OCT intravascular imaging system; St Jude Medical) OCT system to identify ruptured APs (36, 37), which was defined as the presence of fibrous cap discontinuity with cavity formation inside the plaque (38, 39). Analysis of all OCT images was performed by two independent blinded investigators.

### Sample collection and RNA isolation

We obtained peripheral blood from the patients who visited the emergency department within 6 h after the onset of pain; blood samples from patients with plaque rupture identified by OCT examination were used for subsequent analysis, and patients without AMI detected by CAG examination served as the control group. The whole blood samples were collected in ethylenediaminetetraacetic acid (EDTA) tubes before heparin or any contrast agent was administrated. Peripheral blood mononuclear cells (PBMCs) were isolated from all blood samples within 2 h of collection using Ficoll-Paque PREMIUM (Cytiva) according to the manufacturer's instructions.

### RNA sequencing data analysis

Total RNA was isolated from PBMCs with the MagNA Pure Compact System (Roche Diagnostics GmbH, Germany) following the manufacturer's recommendations. Library constructions were performed on VAHTS Total RNA-Seq (H/M/R) Library PrepKit for Illumina and sequencing were performed on Illumina HiSeq 2500 platform according to the manufacturer's specifications. The RNA expression matrix was imported into R software, and the limma package was used for DEGs analysis to identify differentially expressed genes. The identified DEGs were imported into the HiPlot online tool to draw a volcano plot.

### Receiver operating characteristic analysis

To evaluate the capability of hub genes to distinguish patients with plaque rupture from control group, the “pROC” and “ggplot2” package in R studio software were used calculate the area under the curve (AUC) and draw the receiver operating characteristic (ROC) curve (40, 41). To further validate the diagnostic capability of hub genes in plasma for AMI, we selected GSE66360, a data set with larger sample size, for ROC analysis. According to previous studies, 0.7 ≤ AUC < 0.8 represents its acceptable diagnostic power, 0.8 ≤ AUC < 0.9 means excellent diagnostic power, and AUC ≥ 0.9 indicates outstanding evaluation efficacy (42).

### Validate the expression of key genes in foam cells

The human monocytic cell line (THP-1) (ATCC) were grown in complete RPMI-1640 [20% FBS (AU0600); 1% Gluta-max; 1% sodium pyruvate]. Cells were cultured at 37°C in 5% CO_2_ and subcultured at 80%–90% confluence. We used a 100 ng/mL concentration of PMA (Merck) to induce macrophage formation. To induce foam cell formation, the macrophages were incubated with 25 or 50 µg/mL ox-LDL (Yeasen, 20605ES05, China) for 24 h. Foam cells were assessed by Oil Red O Staining kit (Beyotime, C0158S, China).

### Real-time quantitative PCR assay

RNA was extracted from foam cells using the TRIZOL reagent (Invitrogen, CA, United States). Total RNA (500 ng) from foam cells was reverse-transcribed into cDNA using PrimeScript RT Master Mix (TaKaRa, RR036A, China). Real-time qPCR reaction was performed on the QuantStudio™ 5 system (Thermo Fisher Scientific, United States) using TB Green premix Ex Taq (Tli RNaseH Plus, RR420A, Takara) according to the instruction manual. The mRNA expression levels were calculated as the 2^−ΔΔ*Ct*^ value.

## Results

### Datasets grouping information

A total of three GEO datasets numbered GSE28829, GSE163154, and GSE41571 and were included in the current study for analysis of crucial genes and molecular mechanisms associated with plaque progression and rupture. We paired GSE28829 and GSE163154 and performed WGCNA analysis on them as the discovery cohort (Table 1). GSE41571 and GSE163154 were paired for DEGs analysis as a validation cohort (Table 1). Additionally, GSE66360 was used to validate the expression of hub genes in the plasma of AMI patients.

**Table 1:** Datasets grouping information.

Group	GSE number	Samples	Algorithm
Discovery cohort	GSE28829	16 advanced APs and 13 early APs	WGCNA
Discovery cohort	GSE163154	27 IPH APs and 16 non-IPH APs	WGCNA
Validation cohort	GSE163154	27 IPH APs and 16 non-IPH APs	DEGs analysis
Validation cohort	GSE41571	5 ruptured APs and 6 stable APs	DEGs analysis

WGCNA, Weighted Gene Co-Expression Network Analysis; APs, atherosclerotic plaques; IPH, intraplaque hemorrhage; DEGs, Differential Expression Genes.

### Construction of weighted gene co-expression modules in the discovery cohort

**Figure 2:**
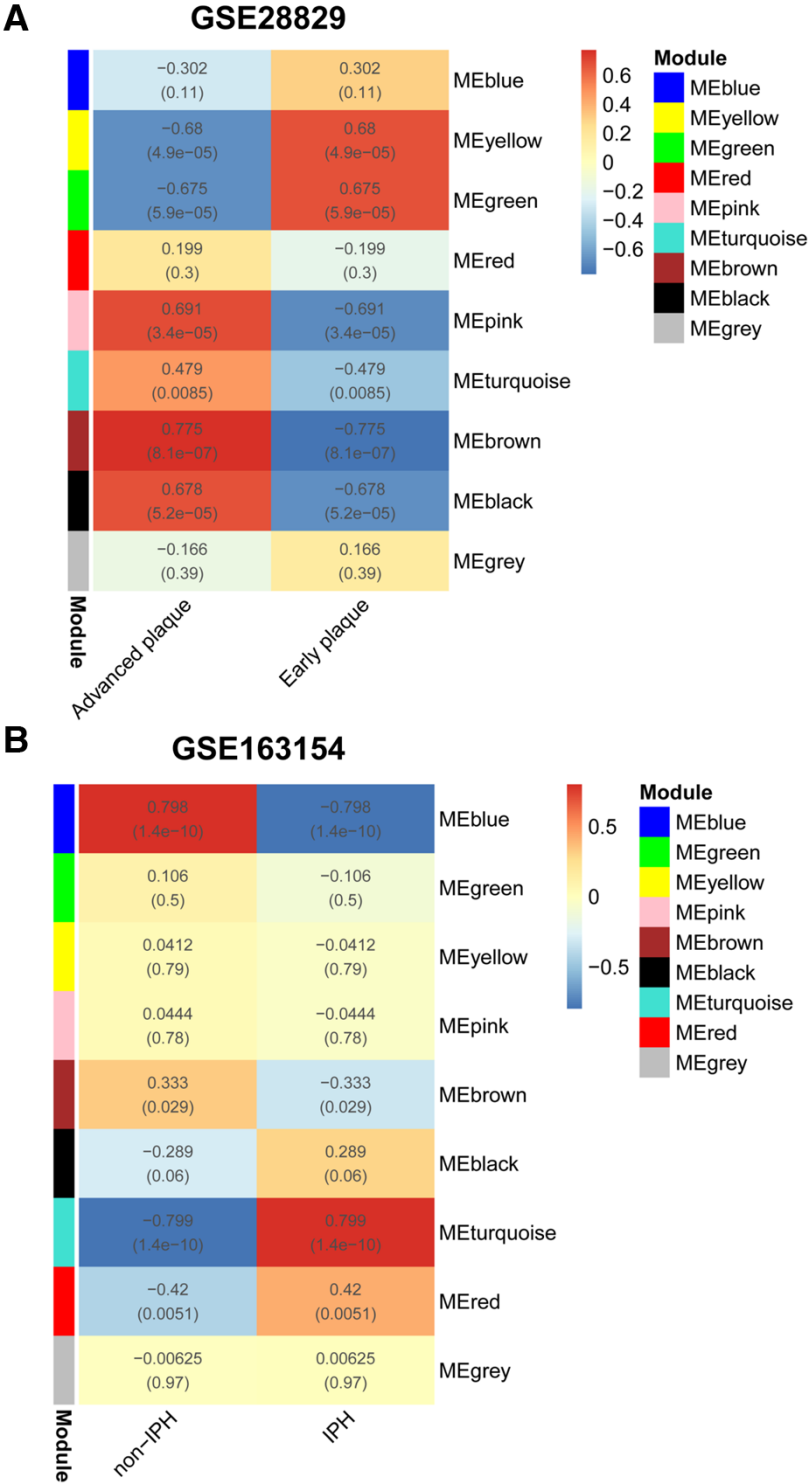
WGCNA. (**A**) Relationship between each co-expression gene module and advanced plaques in GSE28829. Each small lattice contains its corresponding correlation value and *P*-value. (**B**) Relationship between each co-expression gene module and IPH plaques in GSE163154. WGCNA, Weighted Gene Co-Expression Network Analysis; IPH, intraplaque hemorrhage.

**Figure 3:**
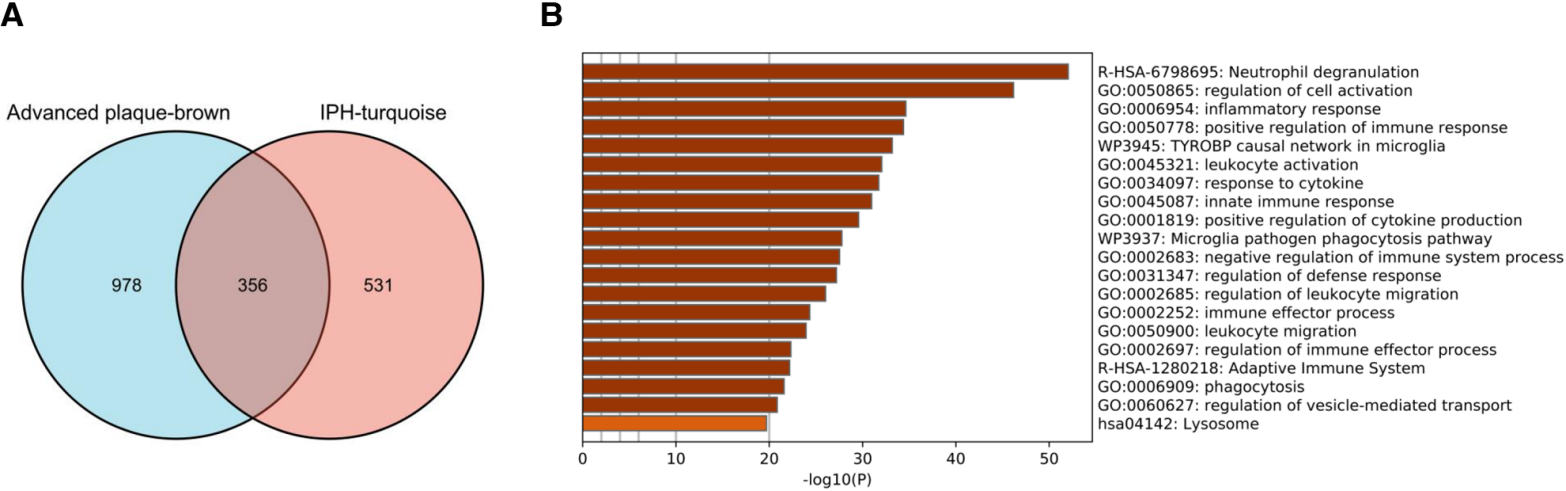
Identification of commonly expressed genes and pathway analysis. (**A**) The shared genes (SET1) between the brown modules of advanced plaques in GSE28829 and the turquoise modules of IPH plaques in GSE163154. (**B**) Signaling pathways significantly enriched in SET1. IPH, intraplaque hemorrhage.

For the analyses of GSE28829 and GSE163154, the soft threshold power *β* = 28 and *β* = 12 were selected for further analysis, respectively (Supplementary Figures S2A, S4A). Next, creating topological overlap matrix (TOM) matrices and modules of genes associated with clinical traits were detected based on the TOM matrices (Supplementary Figures S1, S3). For GSE28829, the Heatmap of module trait relationships showed modules that were highly correlated with APs, with each color representing a specific module (Figure 2A). Among the nine modules identified, three modules “pink”, “brown,” and “black” exhibited significant positive correlations with advanced APs (pink module: r = 0.691, *P* = 3.4 × 10^−05^, brown module: *r* = 0.775, *P* = 8.1 × 10^−07^; black module: *r* = 0.678, *P* = 5.2 × 10^−05^) and the 1,334 genes in the brown module were selected for further analysis (Figures 2A, 3A). Similarly, WGCNA identified nine gene modules in GSE160611 (Figure 2B), and turquoise modules (*r* = 0.799, *P* = 1.4 × 10^−10^) were positively correlated with IPH, including 887 genes (Figures 2B, 3A). The Heatmap shows the correlation between the co-expression modules identified by WGCNA. Red indicates that the two modules are highly correlated, and blue indicates that they are not correlated (Supplementary Figures S4B, S5B). As shown in Supplementary Figure S2B, in GSE28829, there is a relatively high correlation between the brown, turquoise, and black modules. The correlation among the red, turquoise, and black modules was higher in GSE163154 (Supplementary Figure S4B).

### Functional enrichment and pathway analysis

The 356 overlapped genes of the brown module obtained from GSE28829 and turquoise module obtained from GSE163154 were identified as SET-1 (Figure 3A). These genes are strongly associated with the pathogenesis of AP progression and instability. To explore the potential biological functions of SET-1, we conducted functional enrichment and pathway analysis by importing genes in SET-1 into the Metascape database (Figure 3B). The top five most significantly enriched terms were “Neutrophil degranulation,” “regulation of cell activation,” “inflammatory response,” “positive regulation of immune response,” and “TYROBP causal network in microglia.” This indicated that these pathways were closely related to advanced APs and high-risk APs, and the top 10 most significantly enriched pathways are shown in Supplementary Table S1.

### DEGs and related pathways associated with IPH and ruptured APs in the validation cohort

**Figure 4:**
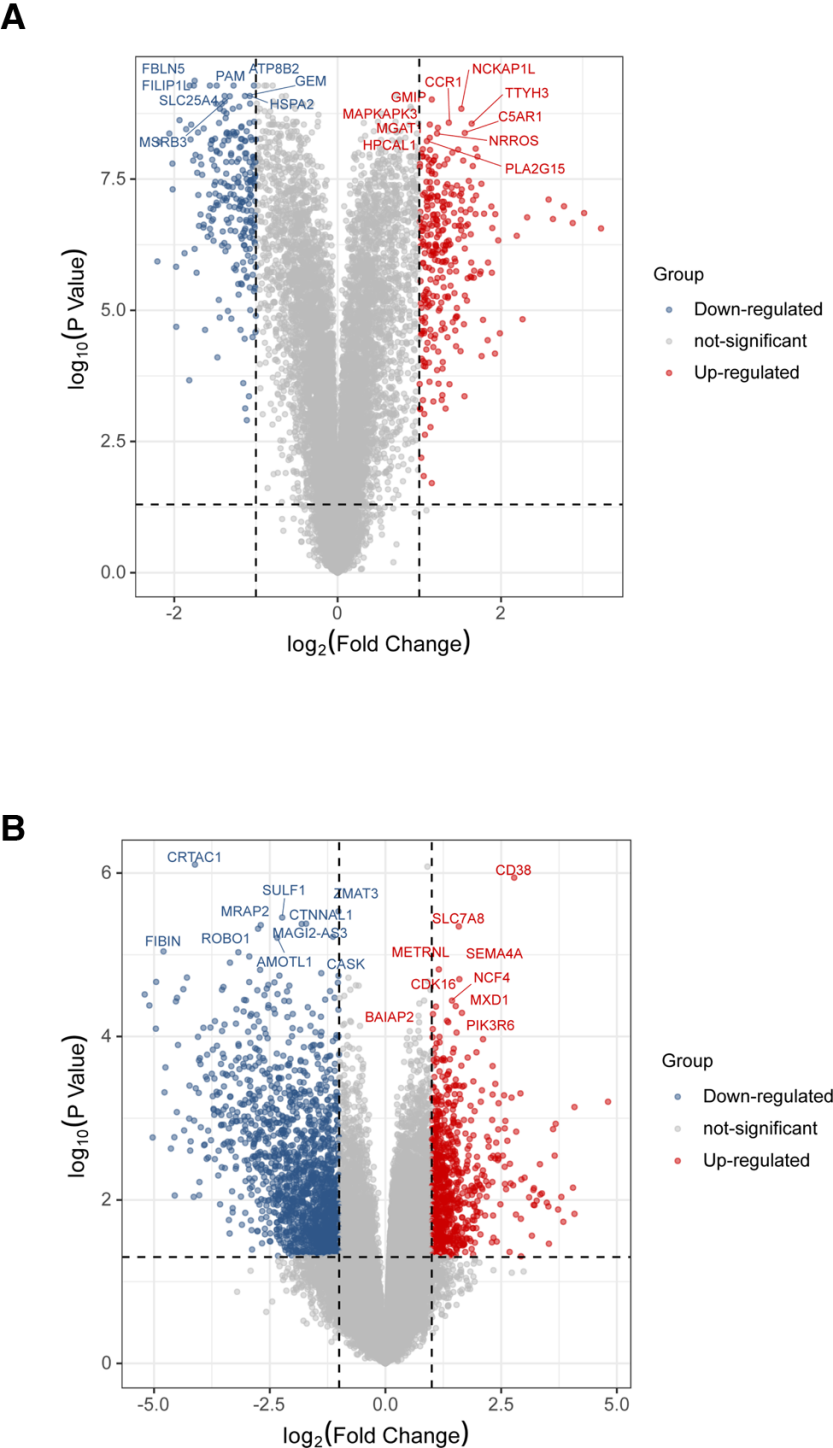
Identification of DEGs in validation cohort. (**A**) Volcanic plot of a total of 499 DEGs in GSE163154 including 270 upregulated and 229 downregulated genes. The y-axis represents the −1og_10_ (*P-*value) and the x-axis represents the gene fold change (log_2_ FC). Blue and red colors dots represent low and high expression, respectively. (**B**) Volcanic map of 1,795 DEGs, including 1,159 downregulated genes and 636 upregulated genes, identified from ruptured AP samples in the GSE41571 dataset. DEGs, Differential Expression Genes.

**Figure 5:**
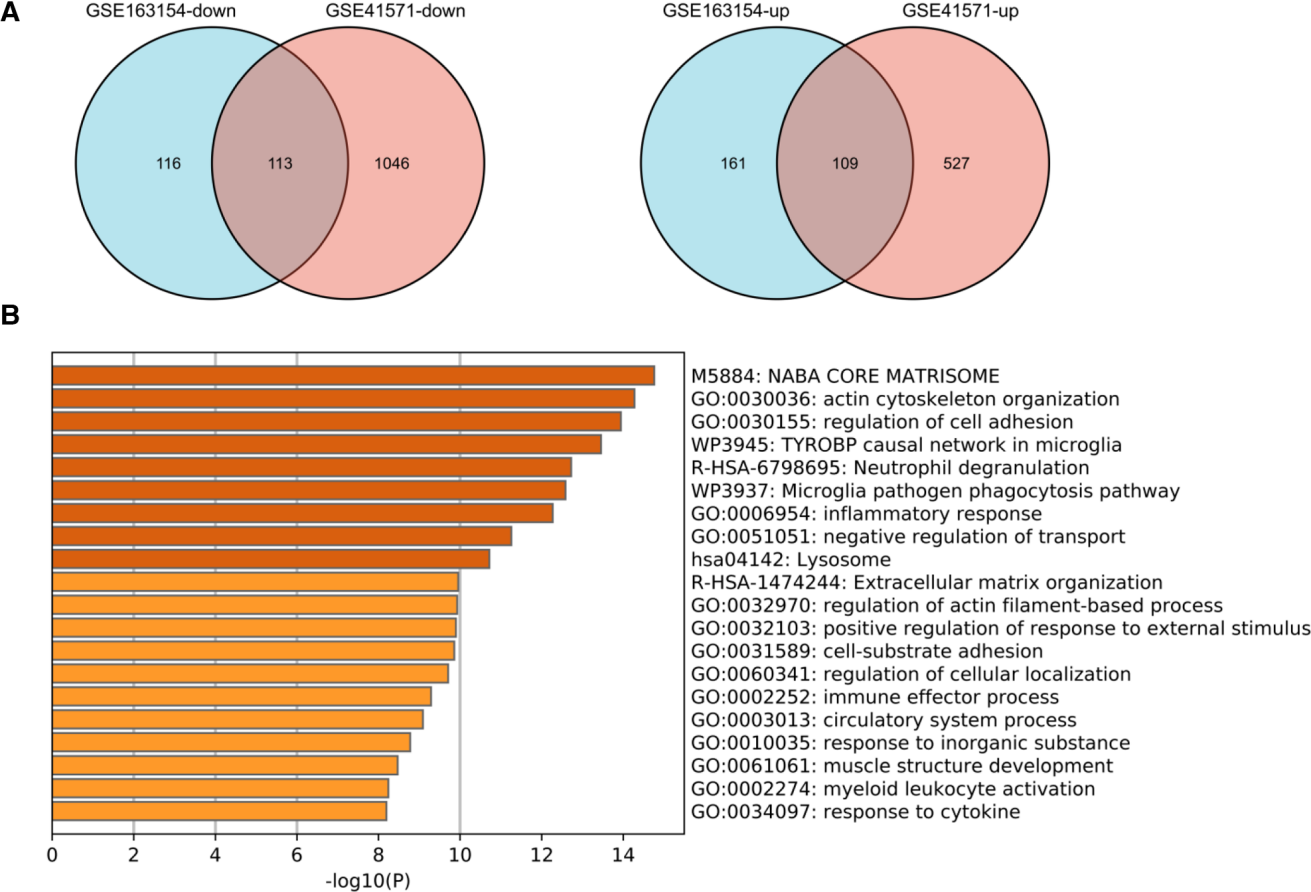
Functional enrichment analysis of shared DEGs in the validation cohort. (**A**) Venn diagram showed the 222 DEGs overlapped between GSE163154 and GSE41571, which were defined as SET2, including 113 downregulated genes and 109 upregulated genes. (**B**) Signaling pathways enriched in SET2. DEGs, Differential Expression Genes.

To validate the analysis results in the discovery cohort, we further searched the GEO database and selected GSE41571 and GSE163154 for DEGs analysis. As shown in the UMAP diagram, IPH AP samples and non-IPH samples are distributed on the right and left, respectively, which indicates that there is a good distinction between the two groups (Supplementary Figure S5A). For GSE163154, a total of 499 DEGs were identified from IPH APs samples based on the gene expression of the non-IPH group, including 270 upregulated genes and 229 downregulated genes (Figures 4A, 5A). GSE41571 contained both ruptured AP and stable AP samples, and UMAP analysis demonstrated a significant discrimination between ruptured AP and stable AP samples (Supplementary Figure S5B). Based on the gene expression of the stable AP group, 1,795 DEGs were identified from ruptured AP samples, including 1,159 downregulated genes and 636 upregulated genes (Figures 4B, 5A). Finally, there were 222 DEGs overlapped between GSE163154 and GSE41571 in the Venn diagram, including 113 downregulated genes and 109 upregulated genes, which were defined as SET-2 (Figure 5A).

Functional enrichment and pathway analysis was performed by importing SET2 obtained from the validation cohort into the Metascape online database (Figure 5B). The top five most significantly enriched terms were “NABA CORE MATRISOME,” “actin cytoskeleton organization,” “regulation of cell adhesion,” “TYROBP causal network in microglia,” and “Neutrophil degranulation” (Figure 5B, Supplementary Table S2). The final results showed that “Neutrophil degranulation” and “TYROBP causal network in microglia” were significantly enriched in both SET-1 and SET-2, suggesting that these two pathways play a crucial role not only in the progression of APs but also in AP rupture (Figures 3B, 5B).

### Identification and functional analysis of hub genes

**Figure 6:**
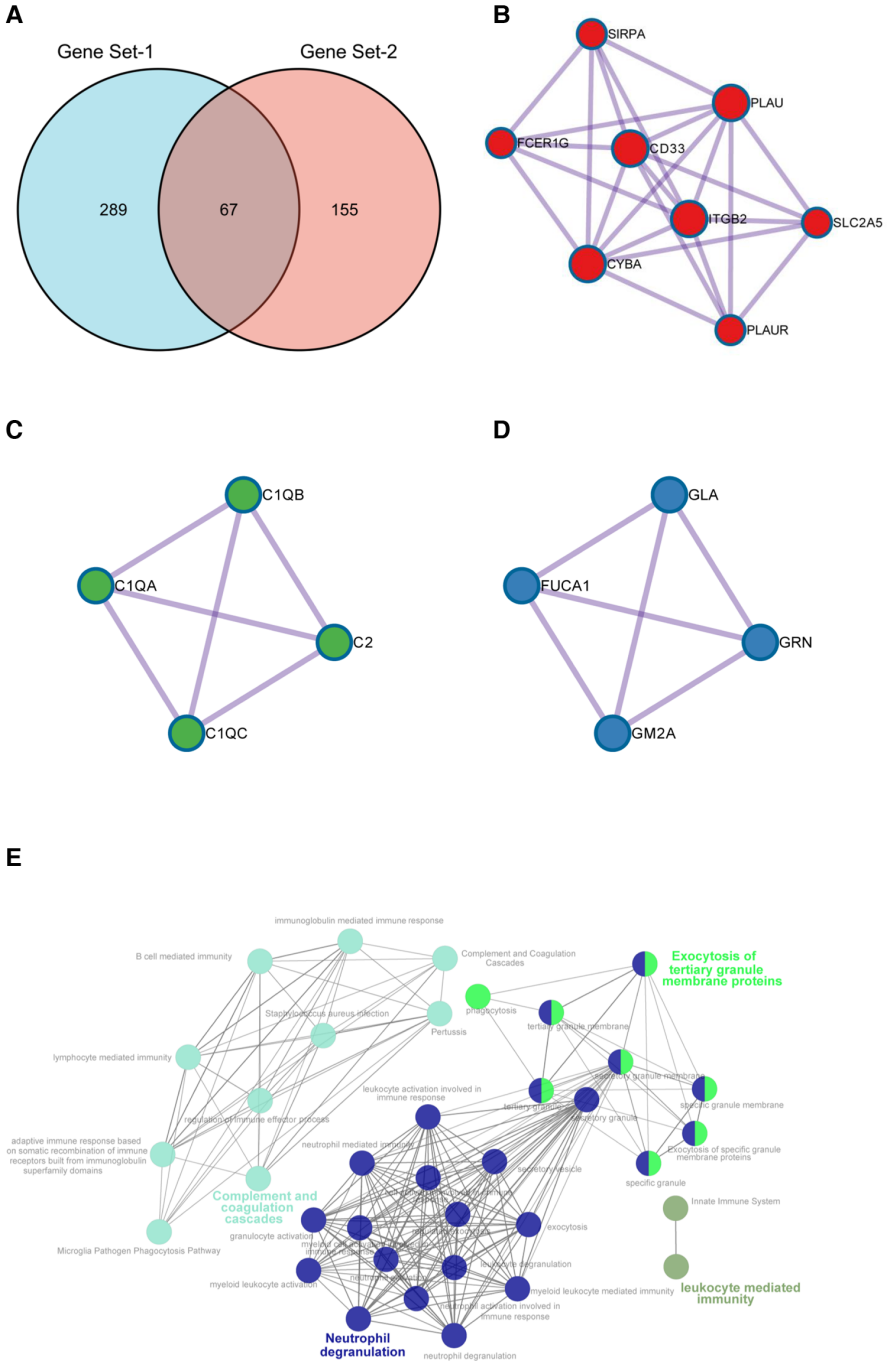
Identification of hub genes. (**A**) Intersection genes of SET1 and SET2. (**B–D**) The three densely connected clusters extracted from shared genes of SET1 and SET2. (**E**) Signal pathway enrichment networks of hub genes constructed by ClueGO. Highlighted words within the network represent the three significantly enriched pathways.

To finally identify reliable hub genes that play key roles in AP progression and rupture, we obtained the intersection genes of SET-1 and SET-2 and introduced them into Metascape for PPI analysis (Figure 6A). The results showed that through the analysis of a total of 67 intersection genes, we obtained 16 hub genes in three densely connected clusters (Figures 6B–D). The results also showed enriched pathways of genes in these three clusters (Supplementary Figure S6, Supplementary Table S3), and “Neutrophil degranulation” was the most significantly enriched term in the first cluster (Figure 6E, Supplementary Figure S6, Supplementary Table S3).

To further validate our conclusions, ClueGO was employed to perform functional enrichment analysis to explore the potential functions of these genes. ClueGO analysis revealed that these hub genes were mainly enriched in pathways in “Neutrophil degranulation,” “Complement and coagulation cascades,” and “Exocytosis of tertiary granule membrane proteins” (Figure 6E).

### The common miRNAs–target genes network

**Figure 7:**
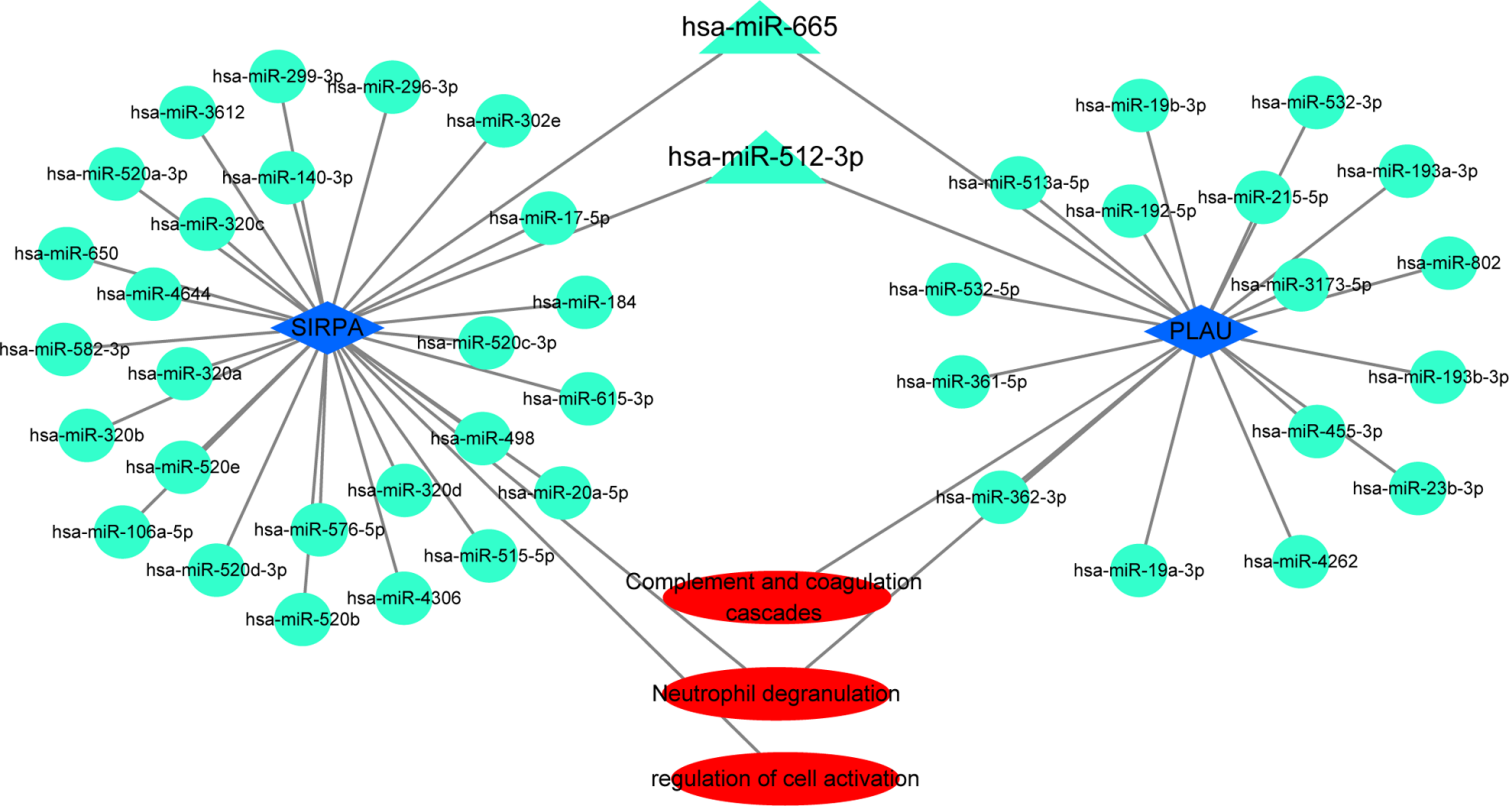
The miRNAs–target genes network. Circles and triangles represent miRNAs, blue diamonds represent hub genes, and triangles indicate two miRNAs with regulatory effects on both hub genes. Red ovals represent signaling pathways.

Hub genes in the first cluster (Figure 6B) were introduced in miRTarBase and ENCORI, and only miRNAs predicted in both databases were used for subsequent analyses. Only *PLAU* and *SIRPA* had common miRNAs available in both databases. These common miRNAs are considered reliable hub gene regulators, and their interaction networks are shown in Figure 6. Among these miRNAs, has-mir-512-3p and has-mir-665 regulated the expression of both *PLAU* and *SIRPA* (Figure 7). In addition, “Neutrophil degranulation” and “Complement and coagulation cascades” are the signaling pathways in which *PLAU* is mainly involved, meanwhile *SIRPA* is mainly related to “Neutrophil degranulation” and “regulation of cell activation.”

### Baseline clinical characteristics

Patients without AMI determined by CAG were included in the control group (*n* = 10), and patients with plaque rupture determined by OCT were included in the plaque rupture group (*n* = 19). The mean age ± SD of the control and the plaque rupture groups was 69 ± 6.976 years and 59.842 ± 11.349 years, respectively. Baseline demographics and clinical characteristics of the control and plaque rupture groups are summarized in Supplementary Table S4. Several parameters showed a significant difference between the two groups.

There were significant differences in four parameters between the two groups: smokers were significantly more common in the plaque rupture group (*P* < 0.001), and plasma levels of BNP, hsCRP, and HB were higher in patients with plaque rupture than in controls (*P* < 0.05).

### Gene expression profiling of patients with plaque rupture

**Figure 8:**
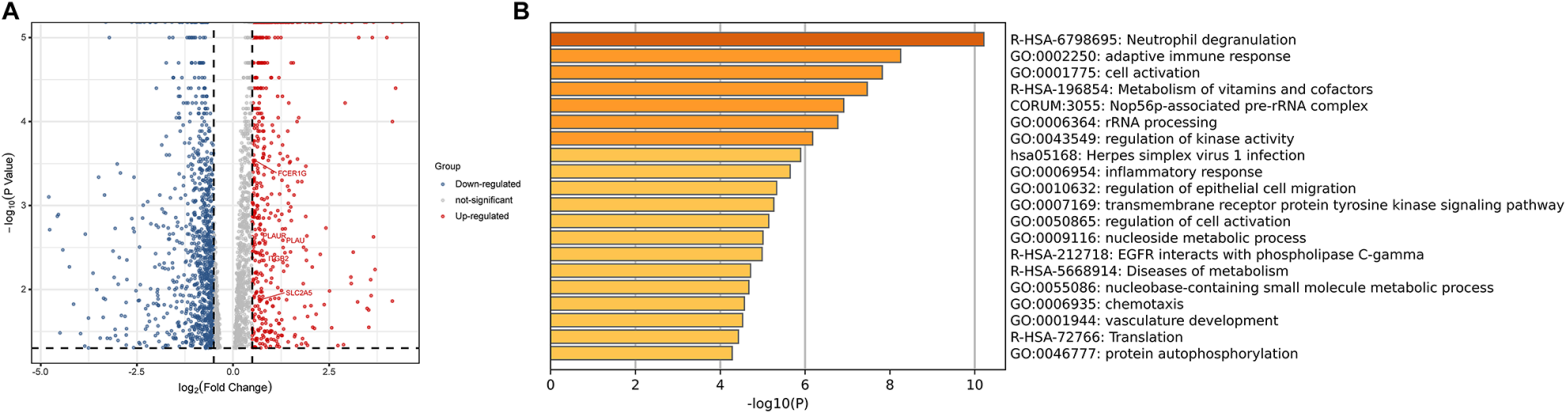
(**A**) The volcanic plot of gene expression profiling of patients with plaque rupture. (**B**) Functional enrichment analysis of DEGs. DEGs, Differential Expression Genes.

Using limma software package for DEGs analysis, set |log FC| ≥ 0.5 and *P-*value < 0.05. According to the gene expression of the control group, 1,215 DEGs were identified from the plasma samples of patients with plaque rupture, including 399 upregulated genes and 816 downregulated genes (Figure 8A). Genes with |log FC| ≥ 1 and *P-*value < 0.05 are presented in Supplementary Figure S5. We further screened 16 hub genes, identified from SET1 andSET2, in the DEGs and observed their expression in the plasma of patients with plaque rupture (Supplementary Table S6). A total of five genes showed significant changes (|log_2_ FC| ≥ 0.5 and *P-*value < 0.05): *PLAU* (log_2_ FC = 1.833836), *PLAUR* (log_2_ FC = 0.703704), *ITGB2* (log_2_ FC = 1.070705), *FERG1G* (log_2_ FC = 0.570931), and *SLC2A5* (log_2_ FC = 0.710641). We imported the filtered differential genes into the “Metascape” database for enrichment analysis, and “Neutrophil degranulation” was the most significantly enriched term (Figure 8B).

### Increased expression of key genes in foam cells

**Figure 9:**
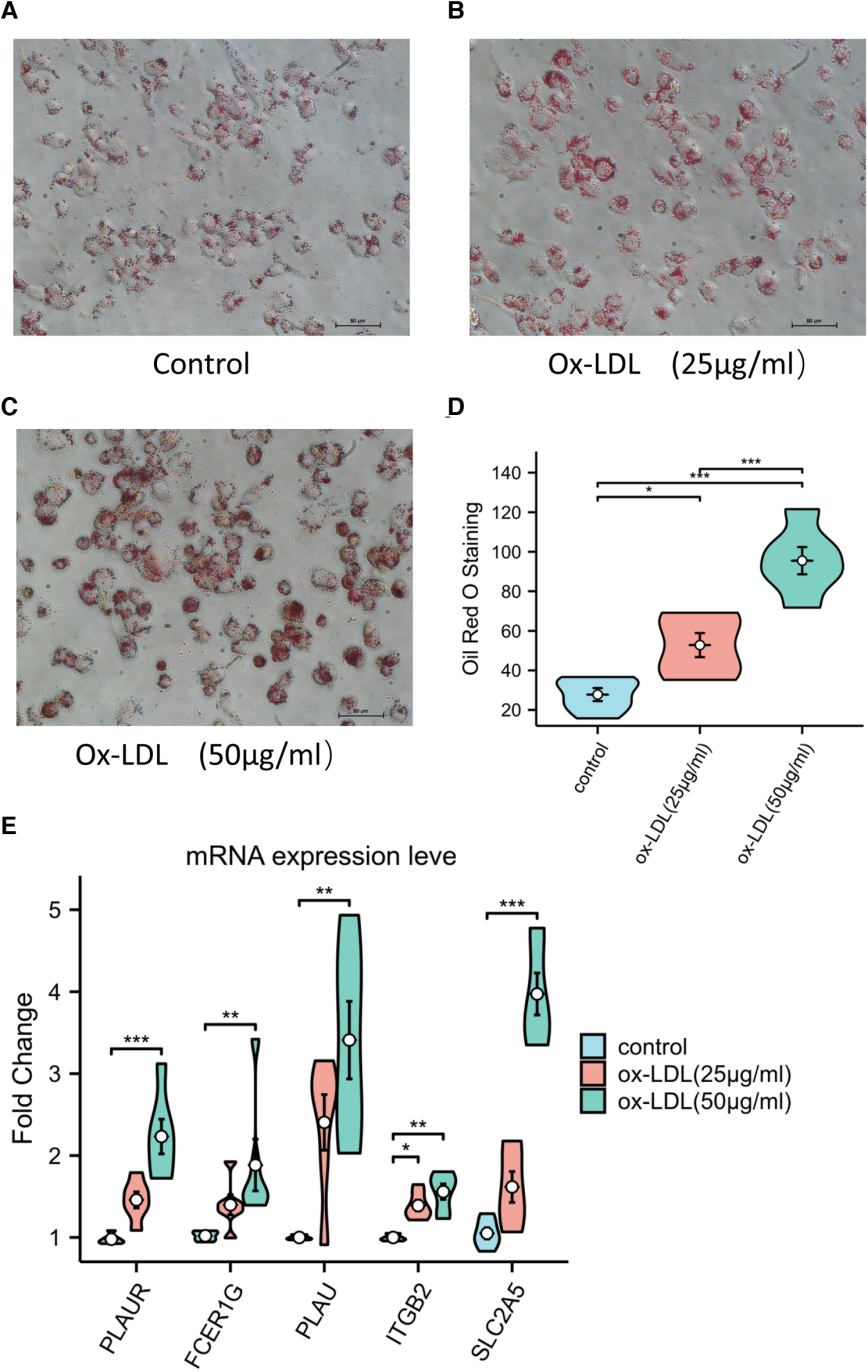
(**A–C**) Representative Oil Red O staining of macrophage. (**D**) Quantitative measurement of Oil Red O staining using ImageJ. (**E**) The expression levels of key genes in ox-LDL-induced macrophage.

After Oil RO staining, a large number of lipid droplets in the cytoplasm were stained red. The quantitative analysis of Oil Red O staining showed the accumulation of lipid in red (Figures 9A–D). The results of the RT-qPCR showed that the expressions level of *PLAUR*, *FCER1G*, *PLAU*, *ITGB2*, and *SLC2A5* were significantly increased in foam cells (Figure 9E). Moreover, as the accumulation of lipid increases, so do the expression level of these genes (Figures 9D,E). This suggests that expressed mRNAs play a fundamental role in the occurrence and development of atherosclerotic plaque.

### ROC curve analyses of the identified hub genes

**Figure 10:**
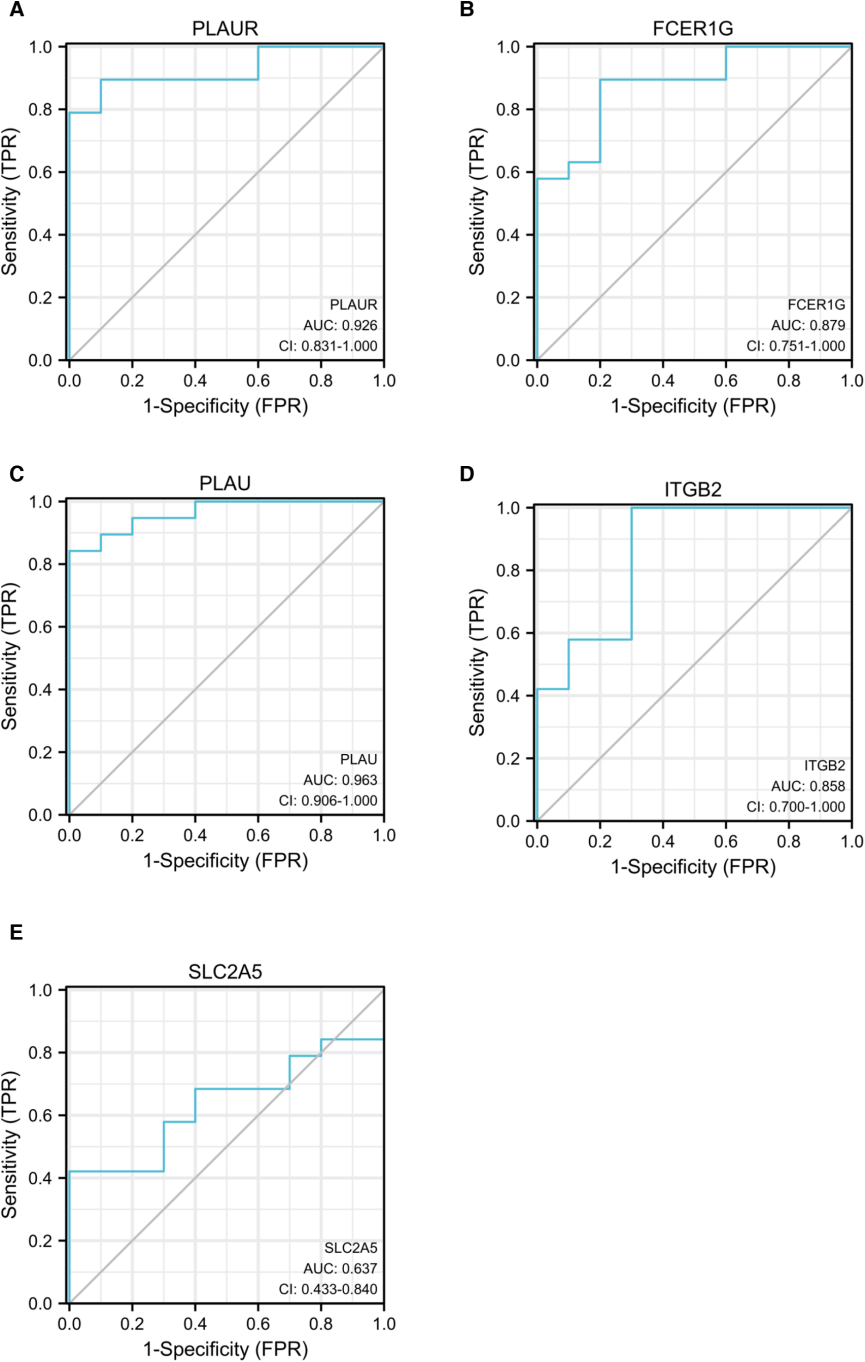
ROC curves for *PLAUR* (**A**), *FCER1G* (**B**), *PLAU* (**C**), *ITGB2* (**D**), and *SLC2A5* (**E**) to assess the capabilities of these genes to discriminate between patients with AP rupture and control group. ROC, receiver operating characteristic; APs, atherosclerotic plaques.

**Figure 11:**
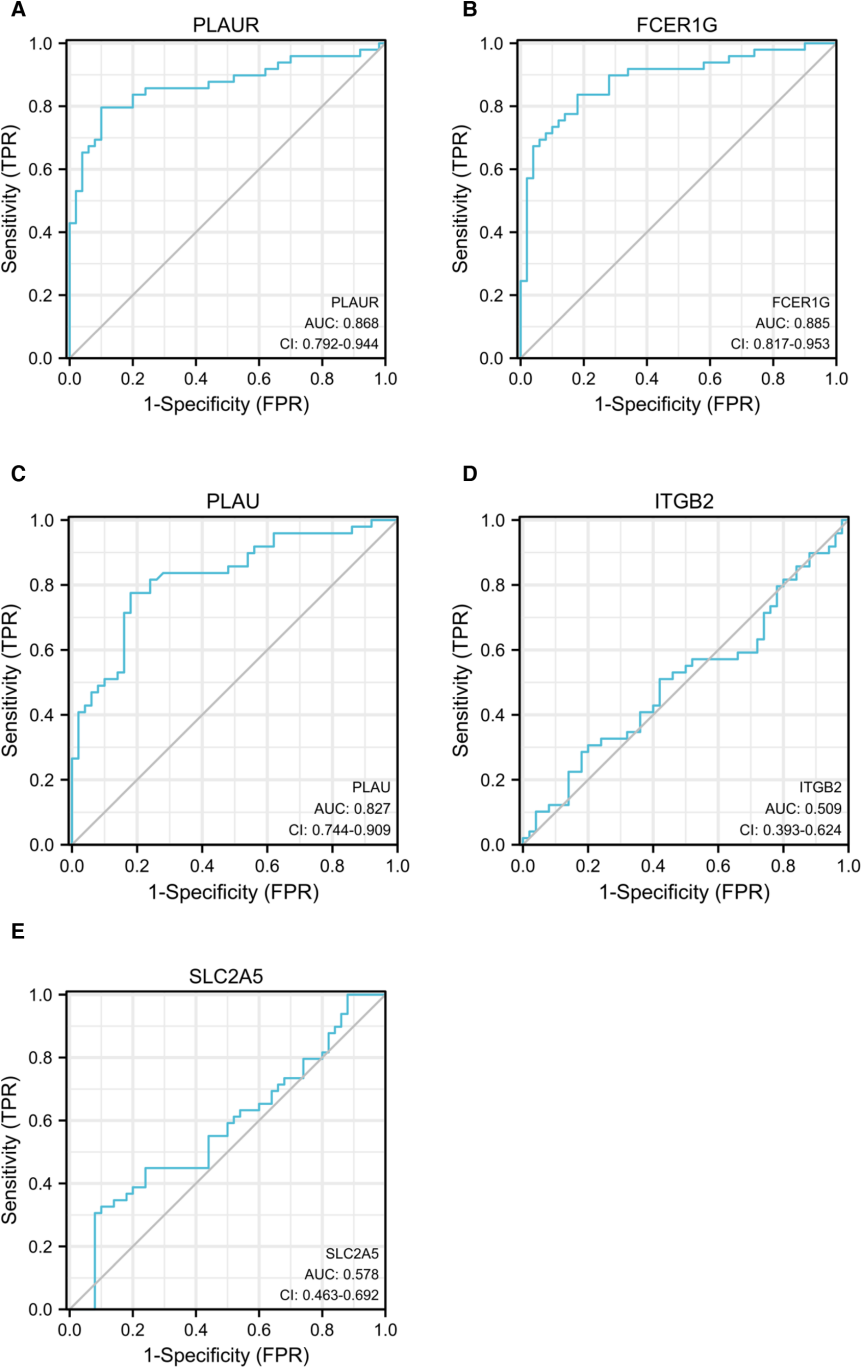
ROC curves of *PLAUR* (**A**), *FCER1G* (**B**), *PLAU* (**C**), *ITGB2* (**D**), and *SLC2A5* (**E**) to evaluate the ability of these genes to discriminate between AMI and control groups. ROC, receiver operating characteristic; AMI, acute myocardial infarction.

ROC curve analysis was conducted to assess the ability of hub genes to discriminate patients with AP rupture from controls. Their ROC curves indicated that the expression of *PLAUR* (AUC = 0.868), *FCER1G* (AUC = 0.879), *PLAU* (AUC = 0.963), *ITGB2* (AUC = 0.858), and *SLC2A5* (AUC = 0.637) could effectively distinguish the patients with plaque rupture and the control group (Figure 10). Moreover, we confirmed the powerful discrimination ability of these genes in GSE66360 with an AUC of 0.868 in *PLAUR* (Figure 11A), AUC of 0.885 in *FCER1G* (Figure 11B), and AUC of 0.827 in *PLAU* (Figure 11C). They also demonstrated strong discriminatory power for AMI patients and control group. However, two other genes, *ITGB2* (AUC = 0.509) (Figure 11D) and *SLC2A5* (AUC = 0.578) (Figure 11E), showed poor discriminatory power. Finally, we identified three genes *PLAUR*, *FCER1G*, and *PLAU* as final hub genes, which we believe represent key factors in the regulation of AP progression and rupture, and may serve as early diagnostic markers of AP instability and rupture.

## Discussion

Most of the acute events that complicate atherosclerosis, such as myocardial infarction and ischemic stroke, are caused by thrombosis, whereas rupture of APs provokes most acute thromboses (3, 43). Rupture of the fibrous cap overlying the surface of the necrotic core of APs exposes highly thrombogenic necrotic core material within the plaque to the blood, triggering the coagulation cascade, resulting in acute thrombosis (44). Elucidating the key processes, based on mechanistic studies or genetic discoveries, involved in the transformation from low-risk to high-risk rupture-prone APs and the subsequent plaque rupture will provide new insights for better prevention, early diagnosis, and targeted therapy in AS-related CVD.

Here, we performed a comprehensive analysis of transcriptomic data related to rupture-prone high-risk APs and ruptured plaques. In the discovery cohort, we explored the co-expression modules with high biological significance related to advanced plaque and rupture-prone high-risk APs (with IPH). To better understand the specific pathobiology of APs progression, we obtained co-expressed genes (SET-1) in key modules and explored the biological processes associated with them. The biological processes involving neutrophil degranulation, cell activation, inflammatory response, and immune response were enriched among SET-1. Neutrophil degranulation was significantly activated during the process of AP progression, and this result is consistent across multiple bioinformatic approaches in both the discovery and validation cohorts.

Neutrophils, as the most abundant leukocyte in blood and critical effector cells of the innate immune system, migrate to the site of tissue inflammation in the early stage of inflammation and may contribute to atherogenesis by amplifying the inflammatory response and mediating the formation of neutrophil extracellular traps (NETs) (45–47). Once activated, neutrophils release phagosomes or secrete antimicrobial and inflammatory proteins that are packed into cytoplasmic granules, a process known as neutrophils degranulation (48). These granules derived from neutrophil degranulation contain proteins essential for regulating neutrophil chemotaxis, antibacterial and recruitment functions, and NET formation of neutrophils (48). Studies have shown that proteinases produced by neutrophil degranulation contribute to plaque instability and may directly participate in AP rupture through NET formation (49–51). Therefore, neutrophil degranulation has major physiological and pathophysiological importance in AP instability and rupture.

To guarantee the accuracy of our findings, we performed DEGs analysis on the data from the validation cohort from another perspective. We overlapped the DEGs obtained from the two datasets in the validation cohort and performed functional analysis on the intersection genes. The results showed that the pathway of neutrophil degranulation was significantly enriched.

In addition, the two sets of genes themselves, SET1 and SET2, may also deserve special attention. A common strategy to uncover hub genes is to consider the intersection of SET1 and SET2 and perform PPI analysis on these intersection genes. We obtained 16 hub genes in three densely connected clusters. Interestingly, the neutrophil degranulation pathway was enriched in both the first and second clusters (Figure 5B, Supplementary Figure S6), which was also demonstrated by ClueGO analysis of the hub genes (Figure 5E). Combined with previous studies, initial results from our analysis revealed that these hub genes and the neutrophil degranulation pathway appear to strongly correlate with the progression and rupture of APs.

Post-transcriptional regulation of mRNA by miRNA plays an essential role in disease progression. We predicted reliable regulatory miRNAs for hub genes by miRTarBase and ENCORI databases and constructed miRNAs–target genes network. This network links miRNAs, hub genes, and signaling pathways to elucidate the molecular mechanism of AP rupture to some extent.

However, improving AS and AP rupture outcomes will require well-functioning plasma markers capable of early diagnosis. To assess whether expression differences of these hub genes in plaques were also present in plasma, we collected whole blood samples from patients with ruptured APs and performed transcriptome sequencing. The utility of hub genes for diagnostic classification was further verified by an external dataset (GSE66360). The three final genes, *PLAUR*, *PLAU*, and *FCER1G*, which were selected for early diagnosis were chosen as they showed potential for diagnostic classification of plaque rupture and AMI, both in our sequencing data and in the external validation set.

*PLAUR* encodes the receptor for urokinase plasminogen activator and, given its role in localizing and promoting plasmin formation, likely influences many normal and pathological processes related to cell-surface plasminogen activation and localized degradation of the extracellular matrix. The interaction between *PLAU* and its cellular receptor *PLAUR*, a key event in cell-surface-associated plasminogen activation, is relevant for cell migration and invasion (52, 53). Previous studies of tissue extracted from aortas and coronary arteries from explanted hearts showed that the content of *PLAUR* gradually increased with the severity of atherosclerosis (53, 54).

A study by Svensson et al. reported that *PLAUR* is highly expressed in monocyte-derived macrophages and symptomatic carotid plaques, and *PLAUR* is predominantly found in ruptured plaque segments (55). Previous studies have shown that *PLAU* is highly expressed in advanced human plaques and rupture-prone areas, and its abundance is related to plaque stability (55, 56). Vaisar et al. showed that elevated *PLAU* activity in plaques leads to basement-membrane protein loss and plaque rupture (57). This view was supported by our observations as the expression levels of *PLAUR* and *PLAU* were significantly increased in both advanced plaques and ruptured plaques, which indicate that the *PLAU*–*PLAUR* system contributes centrally to plaque instability. In addition, the strong diagnostic ability of plasma *PLAUR* (AUC = 0.868) and *PLAU* (AUC = 0.963) levels for AMI also reveals their potential as diagnostic markers.

*FCER1G* is the Fc fragment of the high affinity immunoglobulin E (IgE) receptor Ig, a key molecule involved in allergic responses (58, 59). As a component of the IgE receptor, *FCER1G* mediates allergic inflammatory signaling in mast cells (59). *FCER1G* can bind to the pattern recognition receptors CLEC4E and CLEC4D to form a functional signaling complex in myeloid cells (59, 60). It can also interact with integrins β-2/*ITGB2*-mediated functional ligation in neutrophil activation while also engaging integrins α-2/*ITGA2*-mediated platelet activation (61). However, there have been no reports on the pathological and physiological role of *FCER1G* in AS. Enrichment analysis of this gene revealed that it may be involved in the regulation of atherosclerosis mainly through its involvement in the neutrophil degranulation signaling pathway and may play an important role in plaque instability. Our sequencing results showed that the expression levels of *FCER1G* were significantly increased in the plasma of patients with plaque rupture, which suggests that *FCER1G* (AUC = 0.879) has the potential to be an early diagnostic biomarker for plaque rupture.

Despite substantial advances in the means of prevention and treatment of acute myocardial infarction over the past few decades, the incidence of myocardial infarction has not declined in an increasingly aging population.

We currently know little about the origin and function of many cells in advanced atherosclerotic lesions, and the mechanisms by which they affect plaque stability and plaque rupture leading to myocardial infarction or stroke. The expectation is that the goal of reducing the risk of atherosclerotic plaque rupture or inhibiting atherosclerotic plaque progression can be achieved through our mechanistic studies of atherosclerotic plaque progression and rupture. However, further studies on larger patient populations are needed to definitively demonstrate its value as a prospective biomarker of plaque rupture.

The present investigation scope has many limitations. Further studies with larger patient populations are needed to definitively demonstrate the role that these genes play in atherosclerosis and their value as prospective biomarkers of plaque rupture. In addition, plasma samples from patients with high-risk rupture-prone APs should be obtained, compared with those from patients with ruptured plaques and healthy patients, and further analyzed for the diagnostic efficacy of these genes.

## Conclusion

The present study analyzed altered gene expression profiles in unstable and ruptured APs, identifying that the neutrophil degranulation signaling pathway is closely associated with plaque progression and plaque instability. In addition, the hub genes *PLAUR*, *FCER1G*, and *PLAU* could be used as biomarkers or potential therapeutic targets of APs rupture.

## Data Availability

The datasets presented in this study can be found in online repositories. The names of the repository/repositories and accession number(s) can be found in the article/Supplementary Material.

## Supplementary material

Supplement Figure 1 The cluster dendrogram of co-expression genes in GSE28829.

Supplement Figure 2 (A) soft-threshold power of GSE28829. (B) The heatmap of co-expression modules in GSE28829, red indicates that the two modules are highly correlated, and blue indicates that they are not correlated.

Supplement Figure 3 The cluster dendrogram of co-expression genes in GSE163154.

Supplement Figure 4 (A) soft-threshold power of GSE163154. (B) The heatmap of co-expression modules in GSE163154.

Supplement Figure 5 (A) The UMAP diagram of GSE163154. (B) The UMAP diagram of GSE41571.

Supplement Figure 6 The enriched pathways of hub genes.
