# Supplementary material for: Comprehensive analysis of atherosclerotic plaques reveals crucial genes and molecular mechanisms associated with plaque progression and rupture
Source: Front Cardiovasc Med. 2023 Mar 28;10:951242. doi: 10.3389/fcvm.2023.951242 (PMC10089263; doi:10.3389/fcvm.2023.951242)
Supplement: Supplementary file 1 [file Table1.docx]

| Term | Category | Description | LogP |
| --- | --- | --- | --- |
| R-HSA-6798695 | Reactome Gene Sets | Neutrophil degranulation | -52.0321 |
| GO:0050865 | GO Biological Processes | Regulation of cell activation | -46.1653 |
| GO:0006954 | GO Biological Processes | Inflammatory response | -34.6255 |
| GO:0050778 | GO Biological Processes | Positive regulation of immune response | -34.3672 |
| WP3945 | Wiki Pathways | TYROBP causal network in microglia | -33.1655 |
| GO:0045321 | GO Biological Processes | Leukocyte activation | -32.038 |
| GO:0034097 | GO Biological Processes | Response to cytokine | -31.7222 |
| GO:0045087 | GO Biological Processes | Innate immune response | -30.9544 |
| GO:0001819 | GO Biological Processes | Positive regulation of cytokine production | -29.5595 |
| WP3937 | Wiki Pathways | Microglia pathogen phagocytosis pathway | -27.7609 |

Supplement Table1 Top ten signaling pathways enriched in GS1
