# Supplementary material for: Comprehensive analysis of atherosclerotic plaques reveals crucial genes and molecular mechanisms associated with plaque progression and rupture
Source: Front Cardiovasc Med. 2023 Mar 28;10:951242. doi: 10.3389/fcvm.2023.951242 (PMC10089263; doi:10.3389/fcvm.2023.951242)
Supplement: Supplementary file 2 [file Table2.docx]

| Term | Category | Description | LogP |
| --- | --- | --- | --- |
| M5884 | Canonical Pathways | NABA CORE MATRISOME | -14.7586 |
| GO:0030036 | GO Biological Processes | actin cytoskeleton organization | -14.2736 |
| GO:0030155 | GO Biological Processes | regulation of cell adhesion | -13.9404 |
| WP3945 | WikiPathways | TYROBP causal network in microglia | -13.4525 |
| R-HSA-6798695 | Reactome Gene Sets | Neutrophil degranulation | -12.7213 |
| WP3937 | WikiPathways | Microglia pathogen phagocytosis pathway | -12.5819 |
| GO:0006954 | GO Biological Processes | inflammatory response | -12.267 |
| GO:0051051 | GO Biological Processes | negative regulation of transport | -11.2584 |
| hsa04142 | KEGG Pathway | Lysosome | -10.7191 |
| R-HSA-1474244 | Reactome Gene Sets | Extracellular matrix organization | -9.95566 |

Supplement Table 2 Top ten signaling pathways enriched in GS2
