# Supplementary material for: Comprehensive analysis of atherosclerotic plaques reveals crucial genes and molecular mechanisms associated with plaque progression and rupture
Source: Front Cardiovasc Med. 2023 Mar 28;10:951242. doi: 10.3389/fcvm.2023.951242 (PMC10089263; doi:10.3389/fcvm.2023.951242)
Supplement: Supplementary file 3 [file Table3.docx]

| Color | Cluster | Pathway | Description | Log10(P) |
| --- | --- | --- | --- | --- |
| Red | Cluster-1 | R-HSA-6798695 | Neutrophil degranulation | -14.4 |
| Red | Cluster-1 | M169 | PID INTEGRIN2 PATHWAY | -7.4 |
| Red | Cluster-1 | WP3937 | Microglia pathogen phagocytosis pathway | -6.9 |
| Blue | Cluster-2 | GO:0019377 | glycolipid catabolic process | -9.4 |
| Blue | Cluster-2 | GO:0046466 | membrane lipid catabolic process | -8.2 |
| Blue | Cluster-2 | R-HSA-6798695 | Neutrophil degranulation | -7.2 |
| Green | Cluster-3 | WP545 | Complement activation | -12.7 |
| Green | Cluster-3 | R-HSA-166663 | Initial triggering of complement | -12.6 |
| Green | Cluster-3 | CORUM:6418 | C1q complex | -12.1 |

Supplement Table3 Signaling pathways enriched in hub genes
