# Supplementary material for: Comprehensive analysis of atherosclerotic plaques reveals crucial genes and molecular mechanisms associated with plaque progression and rupture
Source: Front Cardiovasc Med. 2023 Mar 28;10:951242. doi: 10.3389/fcvm.2023.951242 (PMC10089263; doi:10.3389/fcvm.2023.951242)
Supplement: Supplementary file 4 [file Table4.docx]

| Characteristics | | Level | | Overall | Control | Plaque rupture | P value |
| --- | --- | --- | --- | --- | --- | --- | --- |
| n |  | | | 29 | 10 | 19 |  |
| Group (%) | Control | | | 10 (34.48) | 10 (100.00) | 0 (0.00) | <0.0001 |
|  | Plaque rupture | | | 19 (65.52) | 0 (0.00) | 19 (100.00) | |
| Age (mean (SD)) | | |  | 63.000 (10.866) | 69.000 (6.976) | 59.842 (11.349) | 0.0281 |
| Men (%) | | | 0 | 6 (20.69) | 5 (50.00) | 1 (5.26) | 0.019 |
|  | | | 1 | 23 (79.31) | 5 (50.00) | 18 (94.74) |  |
| Women (%) | | | 0 | 23 (79.31) | 5 (50.00) | 18 (94.74) | 0.019 |
|  | | | 1 | 6 (20.69) | 5 (50.00) | 1 (5.26) |  |
| BMI. (kg/m^2^) (mean (SD)) | | |  | 24.466 (2.836) | 24.330 (2.004) | 24.537 (3.238) | 0.8555 |
| Hypertension (%) | | | 0 | 8 (27.59) | 3 (30.00) | 5 (26.32) | 1 |
|  | | | 1 | 21 (72.41) | 7 (70.00) | 14 (73.68) |  |
| Diabetes (%) | | | 0 | 23 (79.31) | 8 (80.00) | 15 (78.95) | 1 |
|  | | | 1 | 6 (20.69) | 2 (20.00) | 4 (21.05) |  |
| HbA1c (median [IQR]) | | |  | 5.800 [5.500, 6.400] | 5.800 [5.550, 6.400] | 5.900 [5.500, 6.400] | 0.9633 |
| Smoking (%) | | | 0 | 12 (41.38) | 8 (80.00) | 4 (21.05) | 0.0077 |
|  | | | 1 | 17 (58.62) | 2 (20.00) | 15 (78.95) |  |
| LDL.C (mean (SD)) | | |  | 3.143 (0.870) | 2.743 (0.871) | 3.353 (0.814) | 0.0719 |
| Uric acid (median [IQR]) | | |  | 344.000 [307.000, 437.000] | 310.000 [275.250, 371.000] | 366.000 [327.500, 446.000] | 0.0939 |
| BNP (median [IQR]) | | |  | 367.500 [145.900, 1422.000] | 115.000 [75.750, 312.500] | 541.300 [274.150, 1591.500] | 0.0218 |
| hsCRP (median [IQR]) | | |  | 2.000 [1.000, 5.360] | 0.110 [0.000, 2.000] | 3.280 [1.575, 7.475] | 0.0076 |
| HB (mean (SD)) | | |  | 142.552 (17.336) | 129.500 (12.878) | 149.421 (15.497) | 0.0017 |
| ACEI.ARB (%) | | | 0 | 7 (24.14) | 5 (50.00) | 2 (10.53) | 0.0568 |
|  | | | 1 | 22 (75.86) | 5 (50.00) | 17 (89.47) |  |
| Statins (%) | | | 0 | 6 (20.69) | 3 (30.00) | 3 (15.79) | 0.6776 |
|  | | | 1 | 23 (79.31) | 7 (70.00) | 16 (84.21) |  |

Supplement Table 4 Baseline Clinical characteristics
