# Supplementary material for: Comprehensive analysis of atherosclerotic plaques reveals crucial genes and molecular mechanisms associated with plaque progression and rupture
Source: Front Cardiovasc Med. 2023 Mar 28;10:951242. doi: 10.3389/fcvm.2023.951242 (PMC10089263; doi:10.3389/fcvm.2023.951242)
Supplement: Supplementary file 5 [file Table5.docx]

Supplement Table 5 Differentially expressed genes

| Gene name | log2FC | P value | Q value |
| --- | --- | --- | --- |
| DEFA1 | 4.391290798 | 0 | 0.000264 |
| DEFA3 | 4.228503688 | 0.00004 | 0.001379 |
| OLAH | 4.155015249 | 0 | 3.85E-06 |
| CDH8 | 4.143168109 | 0.01374 | 0.088686 |
| CRISP2 | 3.999582954 | 0.00001 | 0.000467 |
| AC114730.2 | 3.693505361 | 0.00578 | 0.049238 |
| LOC100421166 | 3.65339239 | 0.00236 | 0.026229 |
| AREG | 3.653267488 | 0 | 6.86E-05 |
| FIGN | 3.622159604 | 0.00001 | 0.000346 |
| BRDT | 3.591406119 | 0.01184 | 0.08006 |
| INSRR | 3.580700766 | 0.00764 | 0.059565 |
| DEFA1B | 3.531490376 | 0.02819 | 0.143442 |
| U82671.8 | 3.487900945 | 0.0168 | 0.100912 |
| SEMA6A | 3.470093106 | 0 | 0.000125 |
| CRISP3 | 3.417089091 | 0 | 6.39E-07 |
| SNORA73A | 3.270200095 | 0.00001 | 0.000548 |
| ADAMTSL3 | 3.240686104 | 0.01407 | 0.090031 |
| PTGIS | 3.125837305 | 0.00359 | 0.035047 |
| CEACAM6 | 3.097696092 | 0 | 6.47E-06 |
| LTF | 3.081529366 | 0 | 2.78E-05 |
| LINC01600 | 2.917238254 | 0 | 0.000111 |
| COL4A1 | 2.914228249 | 0.00006 | 0.001811 |
| MPO | 2.800503348 | 0 | 1.15E-06 |
| CAMP | 2.76503282 | 0 | 4.42E-08 |
| NDNF | 2.727474962 | 0.04652 | 0.199424 |
| KLF14 | 2.56619642 | 0.02857 | 0.144838 |
| LINC00596 | 2.5094458 | 0.01041 | 0.073112 |
| BPI | 2.291815079 | 0 | 1.58E-05 |
| BMX | 2.258542817 | 0 | 7.93E-14 |
| KL | 2.222456415 | 0 | 9.58E-06 |
| SLC25A52 | 2.161672428 | 0.01969 | 0.112594 |
| MCEMP1 | 2.095264499 | 0 | 4.49E-10 |
| ADAMTS2 | 2.073692054 | 0.02814 | 0.14324 |
| LOXHD1 | 2.02136159 | 0 | 3.78E-07 |
| CLEC4D | 1.974463489 | 0 | 3.62E-14 |
| KHDRBS3 | 1.938780463 | 0.02096 | 0.11785 |
| ORM2 | 1.919647495 | 0.0023 | 0.025746 |
| RNASE1 | 1.909327345 | 0.0045 | 0.041288 |
| FAM106DP | 1.900556664 | 0.00338 | 0.033588 |
| ALPK2 | 1.895062697 | 0.00034 | 0.006643 |
| PRRT4 | 1.89207354 | 0.00135 | 0.017795 |
| IL1R2 | 1.881611376 | 0 | 6.78E-11 |
| ARFGEF3 | 1.864744713 | 0.01597 | 0.097389 |
| GGT8P | 1.825381897 | 0.00852 | 0.063999 |
| RNASE3 | 1.78989839 | 0.00052 | 0.008914 |
| KCNE1B | 1.708626684 | 0.00009 | 0.002539 |
| CA4 | 1.695895667 | 0 | 2.06E-09 |
| RCBTB2P1 | 1.676914863 | 0.00028 | 0.005837 |
| PRUNE2 | 1.66546759 | 0.0001 | 0.002699 |
| IL18R1 | 1.662950061 | 0 | 3.38E-08 |
| TMEM52B | 1.652575605 | 0.0467 | 0.199843 |
| TFF3 | 1.603245004 | 0.02192 | 0.120999 |
| GC | 1.600351134 | 0.03643 | 0.169937 |
| ANOS2P | 1.587202151 | 0.00075 | 0.01161 |
| BEND7 | 1.56649119 | 0.00002 | 0.000788 |
| CLEC4E | 1.563178326 | 0 | 9.70E-11 |
| PER1 | 1.55177581 | 0 | 1.34E-05 |
| LINC00937 | 1.541959107 | 0 | 2.33E-09 |
| PLAU | 1.833835798 | 3.14E-03 | 1.21E-07 |
| FCAR | 1.532484719 | 0 | 1.50E-08 |
| SYN2 | 1.515482235 | 0.00151 | 0.019225 |
| FKBP5 | 1.508785659 | 0 | 2.98E-06 |
| DOC2B | 1.502397619 | 0.00104 | 0.014753 |
| KRT5 | 1.494548643 | 0.00357 | 0.034989 |
| SERPINB10 | 1.487419761 | 0.0003 | 0.006144 |
| EREG | 1.470388244 | 0.00031 | 0.006289 |
| CYP4F2 | 1.456853737 | 0 | 2.64E-06 |
| SLC28A3 | 1.456498243 | 0.00066 | 0.010562 |
| ARL4AP2 | 1.44893148 | 0.00006 | 0.001868 |
| RPS3AP43 | 1.440640221 | 0 | 5.52E-05 |
| NACAP3 | 1.438261746 | 0.00094 | 0.013632 |
| RNU12 | 1.433515923 | 0.04202 | 0.186475 |
| F5 | 1.433057198 | 0 | 2.82E-08 |
| PTX3 | 1.423119674 | 0.00013 | 0.003282 |
| ARHGEF17 | 1.419306369 | 0.00059 | 0.009797 |
| SPP1 | 1.41635302 | 0.00026 | 0.005481 |
| MGP | 1.410742636 | 0.00519 | 0.045574 |
| AC000095.9 | 1.393232123 | 0.01326 | 0.086674 |
| CYP4F3 | 1.376219837 | 0 | 4.28E-10 |
| CC2D2B | 1.36886962 | 0 | 2.04E-05 |
| IL1R1 | 1.363310678 | 0 | 2.59E-06 |
| FAM169B | 1.357442827 | 0.00189 | 0.02246 |
| GADD45A | 1.32555551 | 0 | 4.98E-08 |
| NFASC | 1.316872795 | 0.00606 | 0.050933 |
| TPST1 | 1.299987036 | 0 | 4.95E-05 |
| MTND6P3 | 1.298159597 | 0.00028 | 0.005837 |
| GPR15 | 1.288324974 | 0.00019 | 0.00425 |
| DUSP1 | 1.279235835 | 0 | 4.98E-08 |
| GFPT2 | 1.277537259 | 0.03539 | 0.166543 |
| AP000318.2 | 1.271938951 | 0.0005 | 0.008718 |
| SLC27A2 | 1.268211462 | 0.00127 | 0.017196 |
| KIR2DL4 | 1.260475385 | 0.01032 | 0.072725 |
| MGAM2 | 1.25748717 | 0 | 3.66E-06 |
| IFITM10 | 1.245735193 | 0.00001 | 0.000429 |
| APMAP | 1.243762938 | 0 | 3.45E-13 |
| TBC1D8B | 1.241759321 | 0 | 5.35E-05 |
| CDK5R1 | 1.241144943 | 0 | 8.36E-07 |
| IL18RAP | 1.217106551 | 0 | 2.78E-05 |
| A3GALT2 | 1.213757427 | 0.00073 | 0.01142 |
| GCSHP1 | 1.202445654 | 0.02553 | 0.134243 |
| DOCK1 | 1.198093299 | 0.0015 | 0.019153 |
| DDIT4 | 1.194626938 | 0.00003 | 0.001006 |
| SEMA6B | 1.193240983 | 0.00033 | 0.006471 |
| RPS3P6 | 1.188919948 | 0.01276 | 0.084211 |
| IQGAP3 | 1.18782736 | 0.02533 | 0.133579 |
| DZIP1L | 1.184841989 | 0.00015 | 0.003631 |
| ALDOB | 1.175784923 | 0.03523 | 0.166286 |
| MIR646HG | 1.174314081 | 0 | 9.19E-07 |
| FAM95C | 1.167379693 | 0.00001 | 0.000487 |
| Metazoa_SRP | 1.165381272 | 0.0372 | 0.172151 |
| CD163L1 | 1.163940383 | 0.00115 | 0.015944 |
| TMEM92 | 1.163153349 | 0.0095 | 0.068887 |
| SCN9A | 1.160238414 | 0 | 4.93E-07 |
| PLPP3 | 1.160109188 | 0.00005 | 0.001654 |
| DYSF | 1.159579737 | 0 | 2.18E-06 |
| NMNAT2 | 1.130792749 | 0.02323 | 0.1259 |
| CXCL2 | 1.125810602 | 0.04111 | 0.18377 |
| SNORA53 | 1.118003453 | 0 | 8.36E-05 |
| BMP2 | 1.098430679 | 0.0329 | 0.159068 |
| CKAP4 | 1.095891204 | 0 | 2.22E-06 |
| TMEM63C | 1.08545525 | 0.01271 | 0.084038 |
| NRADDP | 1.080039695 | 0.00384 | 0.036805 |
| ITGB2 | 1.070704664 | 0.00449 | 0.001503 |
| TMEM51 | 1.058099015 | 0.03963 | 0.17922 |
| CREM | 1.052314734 | 0.00047 | 0.00839 |
| CEACAM4 | 1.048231263 | 0 | 4.27E-06 |
| LINC01001 | 1.046209609 | 0.00013 | 0.003295 |
| KIAA1462 | 1.038534822 | 0.01584 | 0.097005 |
| PABPN1L | 1.035105557 | 0.00537 | 0.046617 |
| SPATA20P1 | 1.028447389 | 0.01576 | 0.096727 |
| AC078852.1 | 1.021200535 | 0.0004 | 0.007454 |
| ZBTB16 | 1.021109651 | 0.00011 | 0.003015 |
| PGD | 1.014400036 | 0 | 4.22E-09 |
| HCG25 | -1.00127625 | 0.00064 | 0.010305 |
| TRDC | -1.001974547 | 0.00091 | 0.01329 |
| CDK18 | -1.002302094 | 0.00042 | 0.007802 |
| ANKRD36BP2 | -1.002549815 | 0.00181 | 0.021797 |
| ZNF890P | -1.005697079 | 0.02962 | 0.148602 |
| CIB2 | -1.006803974 | 0.01233 | 0.082274 |
| MEST | -1.007190159 | 0.00001 | 0.000326 |
| ARMC9 | -1.007670491 | 0.00281 | 0.029674 |
| PDCD2L | -1.0108613 | 0.00027 | 0.005603 |
| GDF9 | -1.016823438 | 0.01514 | 0.094346 |
| KCNH8 | -1.018370214 | 0.00123 | 0.016739 |
| TLE2 | -1.021020747 | 0 | 0.000216 |
| CORO2B | -1.023726933 | 0.04164 | 0.185241 |
| PDXP | -1.03392642 | 0.02776 | 0.142032 |
| AC024704.2 | -1.036159809 | 0.00088 | 0.013077 |
| SDPR | -1.038457597 | 0.00001 | 0.000323 |
| LINC00336 | -1.043608201 | 0.04559 | 0.197059 |
| PLEKHG5 | -1.043930578 | 0.00397 | 0.037635 |
| SHISA4 | -1.045349637 | 0.01431 | 0.090929 |
| TMEM229B | -1.046780548 | 0.0005 | 0.008728 |
| CDR2L | -1.048804254 | 0.0382 | 0.175028 |
| CXCR2P1 | -1.05186406 | 0.00025 | 0.005281 |
| MSX2P1 | -1.055231063 | 0.00006 | 0.001888 |
| CPA3 | -1.056567619 | 0.00411 | 0.038618 |
| TRIM59 | -1.060009137 | 0.00194 | 0.022883 |
| ANKRD46 | -1.060998285 | 0 | 1.09E-06 |
| SGCE | -1.062583426 | 0.04708 | 0.200736 |
| ZNF462 | -1.067722303 | 0.0035 | 0.034457 |
| GLIPR1L2 | -1.069753823 | 0.02785 | 0.14226 |
| PLLP | -1.070800053 | 0.04075 | 0.182597 |
| RN7SL688P | -1.071481784 | 0.00283 | 0.029882 |
| SSTR3 | -1.071953571 | 0.00037 | 0.007076 |
| ADAMTS10 | -1.073145409 | 0.00002 | 0.000823 |
| LDLRAP1 | -1.075439631 | 0.00002 | 0.000873 |
| DTX1 | -1.081143101 | 0 | 2.12E-05 |
| PTPRK | -1.082442404 | 0.00001 | 0.000526 |
| ADAT3 | -1.08293811 | 0.00151 | 0.019202 |
| ZNF239 | -1.086091837 | 0 | 3.31E-06 |
| P3H3 | -1.087984344 | 0.0075 | 0.058882 |
| RPSAP17 | -1.091909847 | 0.02413 | 0.128909 |
| ZNF620 | -1.092651071 | 0.01494 | 0.093647 |
| AC005329.7 | -1.093359983 | 0.01595 | 0.097323 |
| PPIAP30 | -1.09395323 | 0.00004 | 0.001241 |
| KRT17P2 | -1.094397775 | 0.04373 | 0.191484 |
| IL4I1 | -1.097210826 | 0.00457 | 0.04186 |
| THEM5 | -1.097397743 | 0.03836 | 0.175475 |
| RNF208 | -1.101625473 | 0.01706 | 0.101848 |
| NBL1 | -1.104319024 | 0.04659 | 0.199582 |
| MCC | -1.109146749 | 0.00008 | 0.002368 |
| LINC00920 | -1.111417744 | 0.00348 | 0.034275 |
| IQCC | -1.114225092 | 0.00016 | 0.003871 |
| TIMD4 | -1.116863526 | 0.00167 | 0.020602 |
| STOX1 | -1.121813285 | 0.03403 | 0.162568 |
| GOLGA2P10 | -1.124721958 | 0.0001 | 0.002728 |
| NT5E | -1.129953925 | 0.00001 | 0.000338 |
| KLHL34 | -1.131130042 | 0.00511 | 0.045066 |
| ENHO | -1.13596934 | 0.00795 | 0.061228 |
| TRGC1 | -1.140152469 | 0.00018 | 0.004215 |
| TNS4 | -1.140617415 | 0.01176 | 0.079684 |
| SLC29A2 | -1.143847619 | 0.00001 | 0.000515 |
| ENPP3 | -1.14430074 | 0.00219 | 0.024842 |
| KLRB1 | -1.154450556 | 0.00004 | 0.001294 |
| SIRPG | -1.169230031 | 0 | 1.29E-08 |
| RPS26P6 | -1.170265096 | 0.00364 | 0.035351 |
| NUP62CL | -1.174673995 | 0.00127 | 0.017153 |
| FN1 | -1.180829358 | 0.00772 | 0.060055 |
| ACVR2B | -1.180829584 | 0.01414 | 0.09027 |
| PHGDH | -1.19379332 | 0.00001 | 0.000475 |
| TMEM204 | -1.19994481 | 0 | 0.000128 |
| APOB | -1.200530911 | 0.01715 | 0.102153 |
| FRMD8P1 | -1.207022085 | 0.03652 | 0.170249 |
| CYCSP24 | -1.210428481 | 0.01815 | 0.10609 |
| ZNF285 | -1.217913632 | 0.00006 | 0.001882 |
| TBXA2R | -1.218926846 | 0.00177 | 0.021522 |
| IL17RE | -1.222880051 | 0.00338 | 0.033588 |
| ASB9 | -1.224786444 | 0.02474 | 0.131417 |
| MORC1 | -1.226524395 | 0.04196 | 0.18634 |
| AC104389.16 | -1.235651871 | 0.00473 | 0.042858 |
| GOT2P3 | -1.23856877 | 0.00075 | 0.01157 |
| GULOP | -1.250135237 | 0.02538 | 0.133787 |
| AMN | -1.278594797 | 0.00046 | 0.008299 |
| C11orf1 | -1.288479511 | 0.00006 | 0.001929 |
| CYP46A1 | -1.295383494 | 0.01185 | 0.080095 |
| LOX | -1.297325487 | 0.03103 | 0.153027 |
| GYS2 | -1.30728247 | 0.04302 | 0.189247 |
| CR2 | -1.313374777 | 0 | 4.44E-06 |
| LINC01013 | -1.320004046 | 0.00282 | 0.029756 |
| CLEC4F | -1.324486444 | 0.00903 | 0.066472 |
| OLMALINC | -1.326937804 | 0.00003 | 0.000983 |
| FKBP10 | -1.328166534 | 0.01385 | 0.089075 |
| NBEA | -1.328718119 | 0 | 6.98E-08 |
| NAT8L | -1.333451191 | 0.00946 | 0.068655 |
| DLL1 | -1.355721449 | 0.00082 | 0.01242 |
| LINC01465 | -1.367841165 | 0.01158 | 0.078787 |
| NHLRC4 | -1.377978961 | 0.00005 | 0.001642 |
| LRRC37A9P | -1.387446045 | 0.00276 | 0.029381 |
| CD3EAP | -1.388694906 | 0.00009 | 0.002446 |
| LINC00638 | -1.394161666 | 0 | 0.000124 |
| AC092159.2 | -1.397214628 | 0.02745 | 0.141107 |
| OXTR | -1.398807639 | 0.00042 | 0.007817 |
| ADGRA3 | -1.406650736 | 0.00009 | 0.002611 |
| FLT4 | -1.415064178 | 0.00002 | 0.000931 |
| SPON1 | -1.415691907 | 0.00005 | 0.001665 |
| GPR137C | -1.416333553 | 0.02746 | 0.14112 |
| AL928654.7 | -1.421640579 | 0.0071 | 0.05666 |
| NRXN3 | -1.436336327 | 0.00158 | 0.019855 |
| LYPD2 | -1.441971585 | 0.02502 | 0.132473 |
| GRID2IP | -1.457838643 | 0.00264 | 0.028525 |
| RN7SL180P | -1.45791783 | 0.01399 | 0.089718 |
| PLIN1 | -1.463592614 | 0.00808 | 0.06198 |
| BTF3P7 | -1.468969175 | 0.00961 | 0.069363 |
| NPAS2 | -1.472381111 | 0.00009 | 0.002482 |
| APOE | -1.487668746 | 0.00139 | 0.018158 |
| PCGF2 | -1.494024533 | 0.03745 | 0.172738 |
| APOA1 | -1.509224213 | 0.00063 | 0.010232 |
| PF4V1 | -1.525267879 | 0.00328 | 0.032959 |
| AC092566.1 | -1.532806428 | 0.00817 | 0.062316 |
| U62631.5 | -1.53447818 | 0.03846 | 0.175751 |
| IGF2 | -1.548605172 | 0.00003 | 0.001211 |
| MMP28 | -1.55410304 | 0.00217 | 0.024733 |
| COL5A2 | -1.555448214 | 0.00003 | 0.001218 |
| MMP11 | -1.564414651 | 0.00001 | 0.000584 |
| GPX2 | -1.566730876 | 0.00553 | 0.047646 |
| XIRP2 | -1.573672637 | 0.00482 | 0.04333 |
| SERPINE2 | -1.588953465 | 0 | 2.19E-05 |
| FAM167B | -1.619636957 | 0.0158 | 0.096867 |
| AC064834.2 | -1.629201803 | 0.00081 | 0.012276 |
| PDE9A | -1.656197522 | 0.00001 | 0.000392 |
| LINC00471 | -1.660875981 | 0.00968 | 0.06979 |
| GPR25 | -1.665134525 | 0.00371 | 0.035826 |
| ATP5A1P8 | -1.674676964 | 0.00251 | 0.027452 |
| SLC25A24P1 | -1.690225508 | 0.04623 | 0.198928 |
| AP001065.15 | -1.712543383 | 0.01923 | 0.110797 |
| KLHL31 | -1.729590039 | 0.00025 | 0.00534 |
| FBXO15 | -1.736710415 | 0.00002 | 0.000746 |
| NDUFA4L2 | -1.742705499 | 0.04657 | 0.199514 |
| ADAM29 | -1.755729829 | 0.04631 | 0.199013 |
| ATP8A2 | -1.764267553 | 0 | 1.01E-06 |
| GDF10 | -1.766955854 | 0.00563 | 0.048313 |
| HSPD1P5 | -1.787900636 | 0.0152 | 0.094559 |
| GEM | -1.800218267 | 0.02148 | 0.119591 |
| IGSF11 | -1.809536589 | 0.00175 | 0.021308 |
| TMEM136 | -1.821114517 | 0.04911 | 0.206209 |
| ASMT | -1.830556033 | 0.00551 | 0.047514 |
| KIAA1217 | -1.844220366 | 0.0011 | 0.015429 |
| SERPINA3 | -1.851668286 | 0.00019 | 0.004264 |
| E2F6P4 | -1.903818439 | 0.00075 | 0.011621 |
| S100A1 | -1.924853079 | 0.04152 | 0.184908 |
| AC140725.4 | -1.938010665 | 0.01556 | 0.095898 |
| C5orf17 | -1.943347433 | 0.00181 | 0.021839 |
| PBK | -1.946272831 | 0.0153 | 0.094917 |
| RASL10B | -1.947615638 | 0.01297 | 0.085269 |
| AC022210.2 | -1.949454467 | 0.02713 | 0.14002 |
| ANKRD29 | -1.957464584 | 0.03318 | 0.159907 |
| PRSS22 | -1.958606571 | 0.01001 | 0.07119 |
| PRSS35 | -1.971109622 | 0.01079 | 0.075023 |
| CHAD | -1.99264104 | 0.01473 | 0.092742 |
| GDF15 | -1.993722816 | 0.00004 | 0.001503 |
| TDRD12 | -1.997345749 | 0.00171 | 0.020931 |
| AC004076.7 | -2.012889589 | 0.00374 | 0.036086 |
| CCDC110 | -2.017185026 | 0.02245 | 0.122818 |
| CA6 | -2.134223669 | 0.00165 | 0.020441 |
| MPPED2 | -2.152578679 | 0 | 5.18E-06 |
| THEGL | -2.160088395 | 0 | 0.000112 |
| EIF3LP2 | -2.173580992 | 0.02397 | 0.12831 |
| ZNF969P | -2.198811218 | 0.02 | 0.113823 |
| AP000282.2 | -2.216515678 | 0.01555 | 0.095859 |
| SPINK2 | -2.218590282 | 0.0009 | 0.013212 |
| POTEI | -2.247090821 | 0.04699 | 0.200537 |
| LINC00535 | -2.270522602 | 0.01692 | 0.101241 |
| A4GALT | -2.272465306 | 0.02966 | 0.148673 |
| GJA3 | -2.308837494 | 0 | 1.13E-05 |
| VANGL2 | -2.35039829 | 0.00141 | 0.018335 |
| ROBO1 | -2.398879929 | 0 | 2.45E-05 |
| TLX2 | -2.421047317 | 0.04457 | 0.194016 |
| LINC00689 | -2.432100031 | 0.00312 | 0.031881 |
| WDR93 | -2.437756332 | 0.01346 | 0.087472 |
| CABP7 | -2.443954314 | 0.04283 | 0.188617 |
| IL23R | -2.448601674 | 0 | 6.43E-06 |
| SERHL | -2.463249802 | 0.01358 | 0.087961 |
| NOG | -2.505547658 | 0 | 4.89E-14 |
| LYPD5 | -2.574493465 | 0.00046 | 0.008214 |
| AC091493.2 | -2.584703078 | 0.04924 | 0.206611 |
| AC004791.2 | -2.595267213 | 0.00187 | 0.022266 |
| GRM4 | -2.629936908 | 0.00143 | 0.018483 |
| TRDV2 | -2.633746242 | 0 | 1.28E-06 |
| CYP21A2 | -2.760095598 | 0.00445 | 0.040979 |
| AC079610.2 | -2.769834121 | 0.02934 | 0.147508 |
| PTPN20CP | -2.777443871 | 0.03484 | 0.164966 |
| KCNG3 | -2.777628708 | 0.04592 | 0.198038 |
| SLC7A4 | -2.809019616 | 0.02088 | 0.117462 |
| FLJ30679 | -2.903515101 | 0.02037 | 0.115362 |
| PGC | -2.92809972 | 0.00041 | 0.007567 |
| PLEKHG6 | -2.986286443 | 0.00865 | 0.06462 |
| C1orf234 | -3.011964228 | 0.00032 | 0.006349 |
| ANGPT4 | -3.056882765 | 0.03541 | 0.166549 |
| SLC22A31 | -3.092229512 | 0.00717 | 0.057123 |
| CARD10 | -3.12029512 | 0.00348 | 0.034274 |
| RN7SL597P | -3.194156863 | 0.03826 | 0.175219 |
| TMEM132C | -3.216518125 | 0.00001 | 0.000545 |
| GTF2IP7 | -3.341029106 | 0.00075 | 0.011582 |
| IGDCC3 | -3.366463103 | 0.01272 | 0.084046 |
| OR1J4 | -3.377033339 | 0.02147 | 0.119545 |
| XIST | -3.639759973 | 0.00638 | 0.052721 |
| AC005307.1 | -3.759697653 | 0.02189 | 0.120861 |
| MAEL | -3.840884297 | 0.00219 | 0.024844 |
| AC072052.7 | -3.854990932 | 0.04717 | 0.200989 |
| RPL21P12 | -3.963929533 | 0.03345 | 0.160801 |
| FAM166A | -4.146076986 | 0.01494 | 0.093647 |
| AC226118.1 | -4.254879838 | 0.00537 | 0.046617 |
| AKR1B10 | -4.767093666 | 0.00194 | 0.022883 |
| CYP26B1 | -4.78934331 | 0.00079 | 0.012048 |
