# Supplementary material for: Comprehensive analysis of atherosclerotic plaques reveals crucial genes and molecular mechanisms associated with plaque progression and rupture
Source: Front Cardiovasc Med. 2023 Mar 28;10:951242. doi: 10.3389/fcvm.2023.951242 (PMC10089263; doi:10.3389/fcvm.2023.951242)
Supplement: Supplementary file 6 [file Table6.docx]

| Gene name | log2FC | P value | Q value |
| --- | --- | --- | --- |
| SIRPA | 0.463679 | 0.000485 | 0.00855 |
| FCER1G | 0.570931 | 0.00029 | 0.005916 |
| CYBA | 0.242406 | 0.000827 | 0.012485 |
| PLAUR | 0.703704 | 1.94E-03 | 0.000129 |
| PLAU | 1.833836 | 3.14E-03 | 1.21E-07 |
| CD33 | 0.181392 | 0.065111 | 0.247807 |
| ITGB2 | 1.070705 | 4.49E-03 | 0.001503 |
| SLC2A5 | 0.710641 | 0.013232 | 0.086531 |
| C1QB | -0.39653 | 0.555214 | 0.840308 |
| C1QA | -0.44329 | 0.42636 | 0.736742 |
| C1QC | 0.114064 | 0.523582 | 0.820227 |
| GLA | 0.223587 | 0.015884 | 0.097136 |
| FUCA1 | -0.24179 | 0.544142 | 0.83168 |
| GM2A | 0.326895 | 0.002418 | 0.026665 |
| GRN | 0.453519 | 0.000512 | 0.00886 |

Supplement Table 6 Expression differences of hub genes
