# Supplementary figures and images for: Comprehensive analysis of atherosclerotic plaques reveals crucial genes and molecular mechanisms associated with plaque progression and rupture
Source: Front Cardiovasc Med. 2023 Mar 28;10:951242. doi: 10.3389/fcvm.2023.951242 (PMC10089263; doi:10.3389/fcvm.2023.951242)

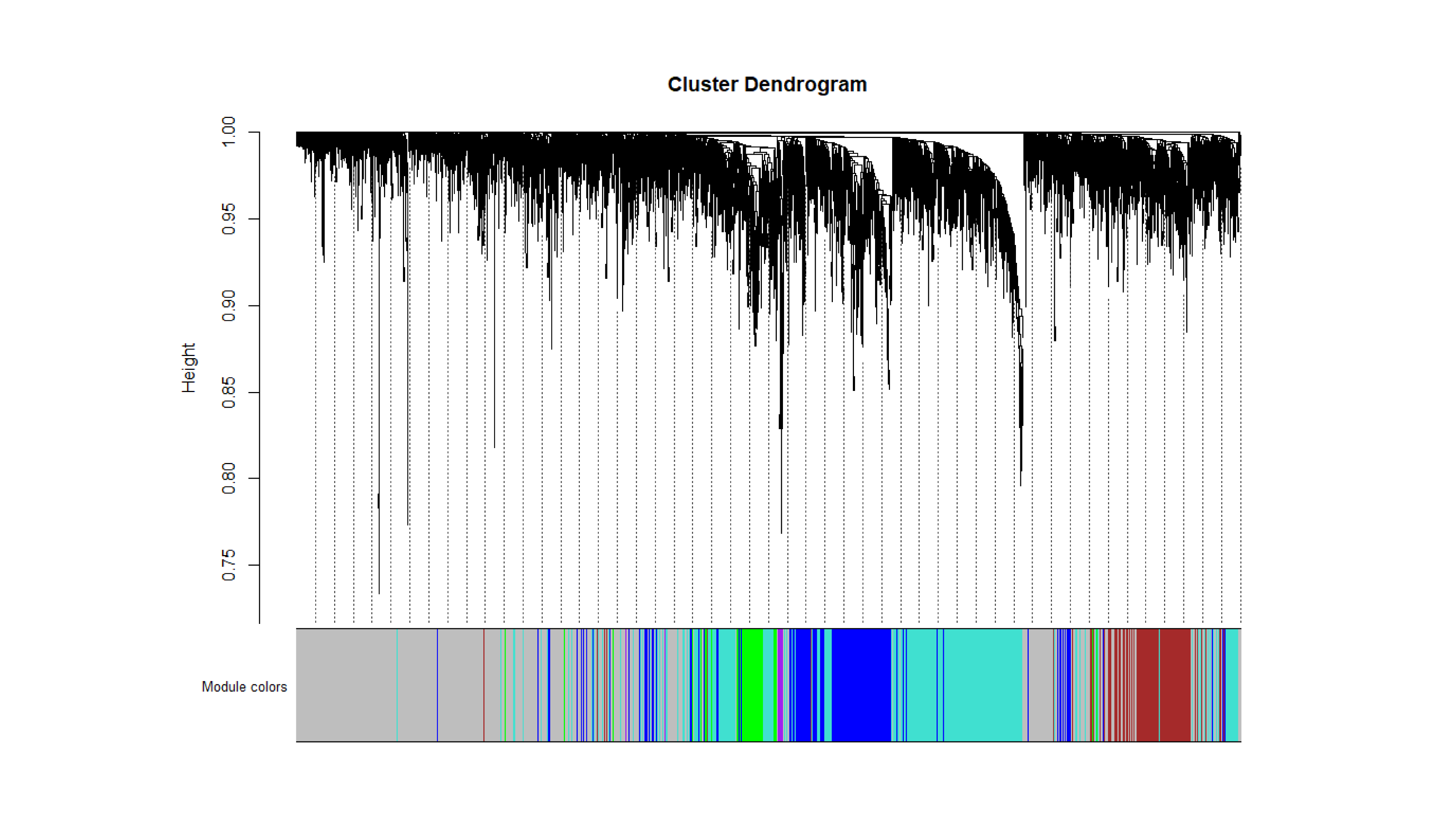

Supplement: Supplement Figure 1 — The cluster dendrogram of co-expression genes in GSE28829. [file Image1.tif]

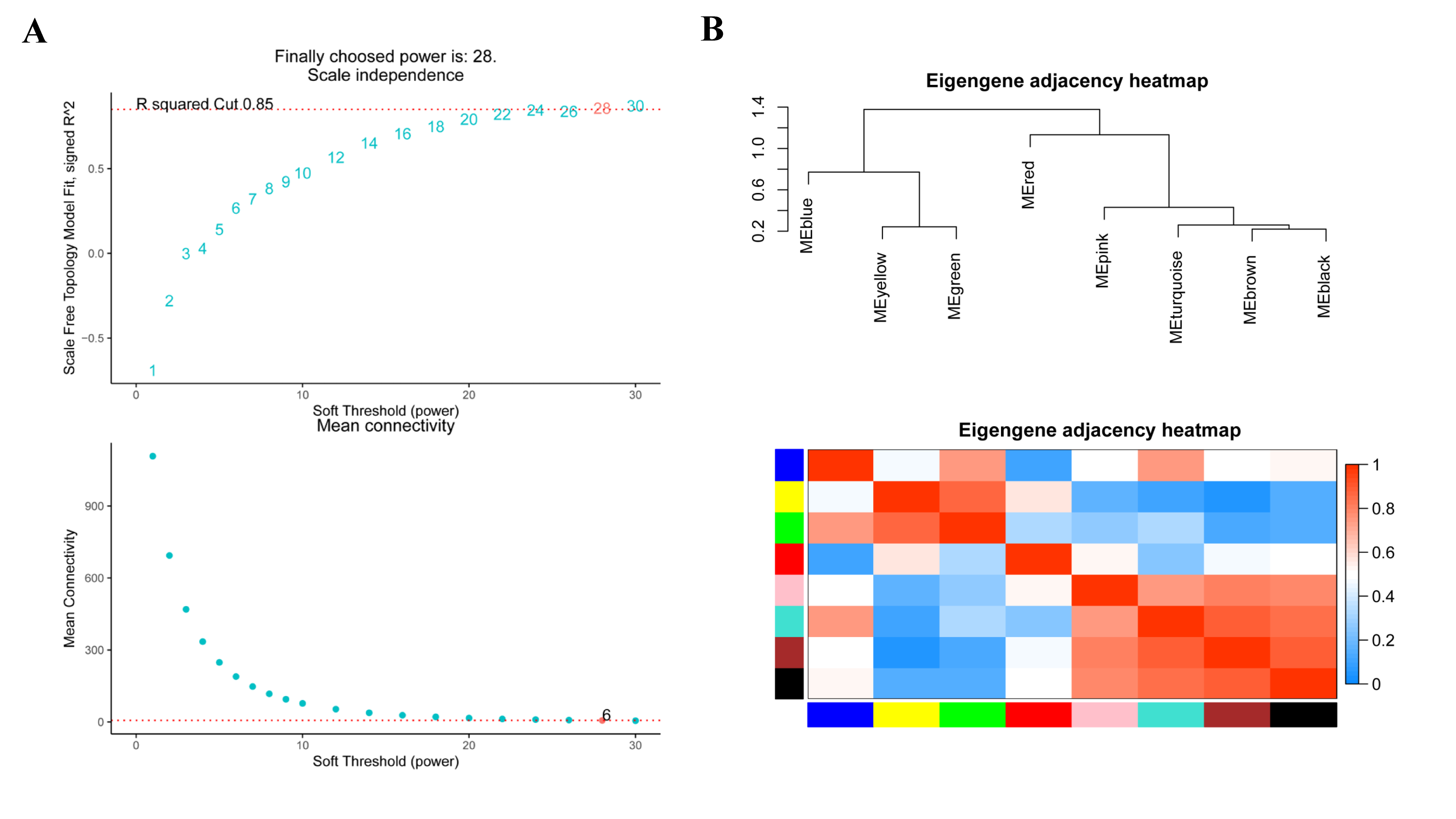

Supplement: Supplement Figure 2 — (A) soft-threshold power of GSE28829. (B) The heatmap of co-expression modules in GSE28829, red indicates that the two modules are highly correlated, and blue indicates that they are not correlated. [file Image2.tif]

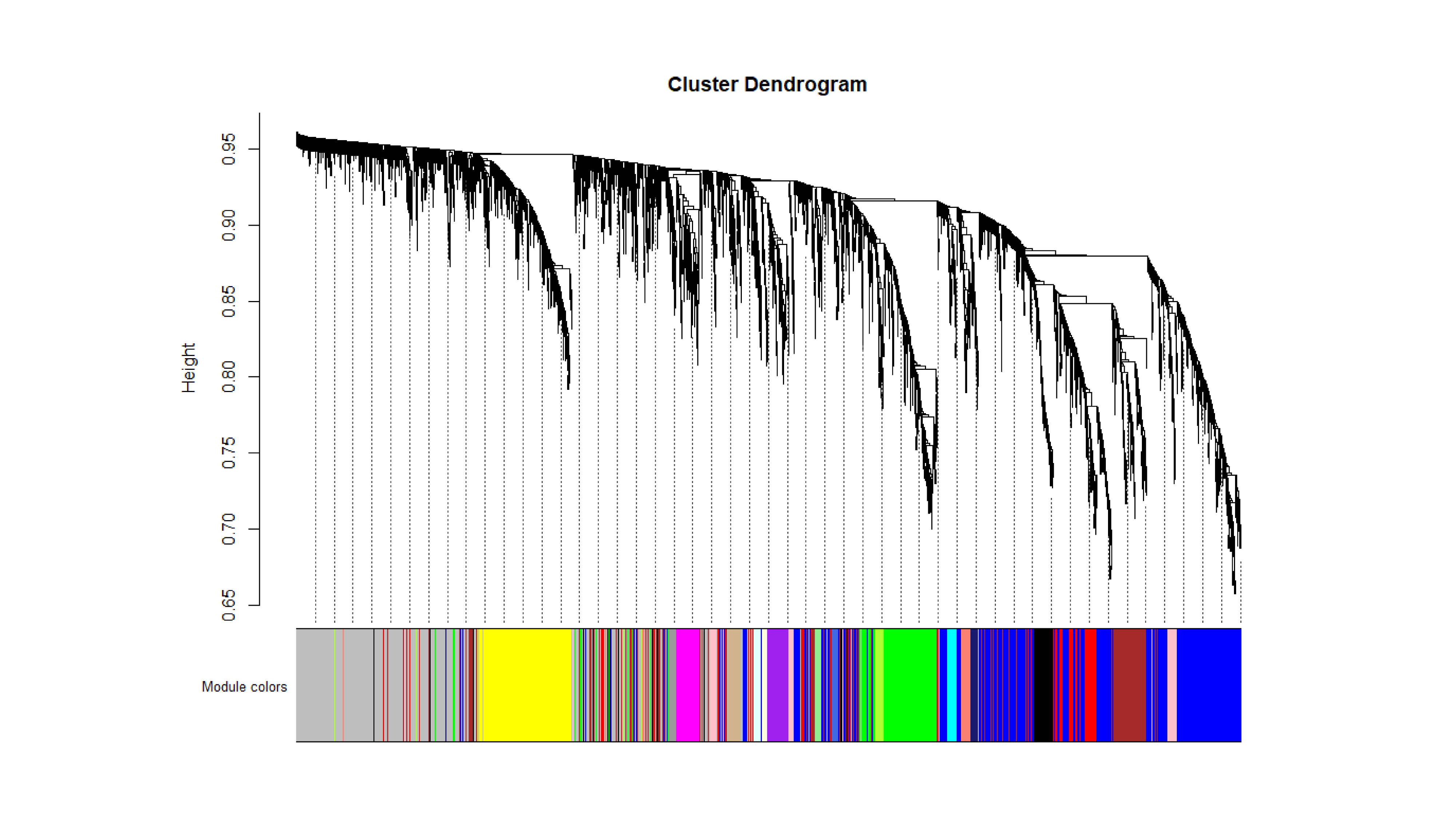

Supplement: Supplement Figure 3 — The cluster dendrogram of co-expression genes in GSE163154. [file Image3.tif]

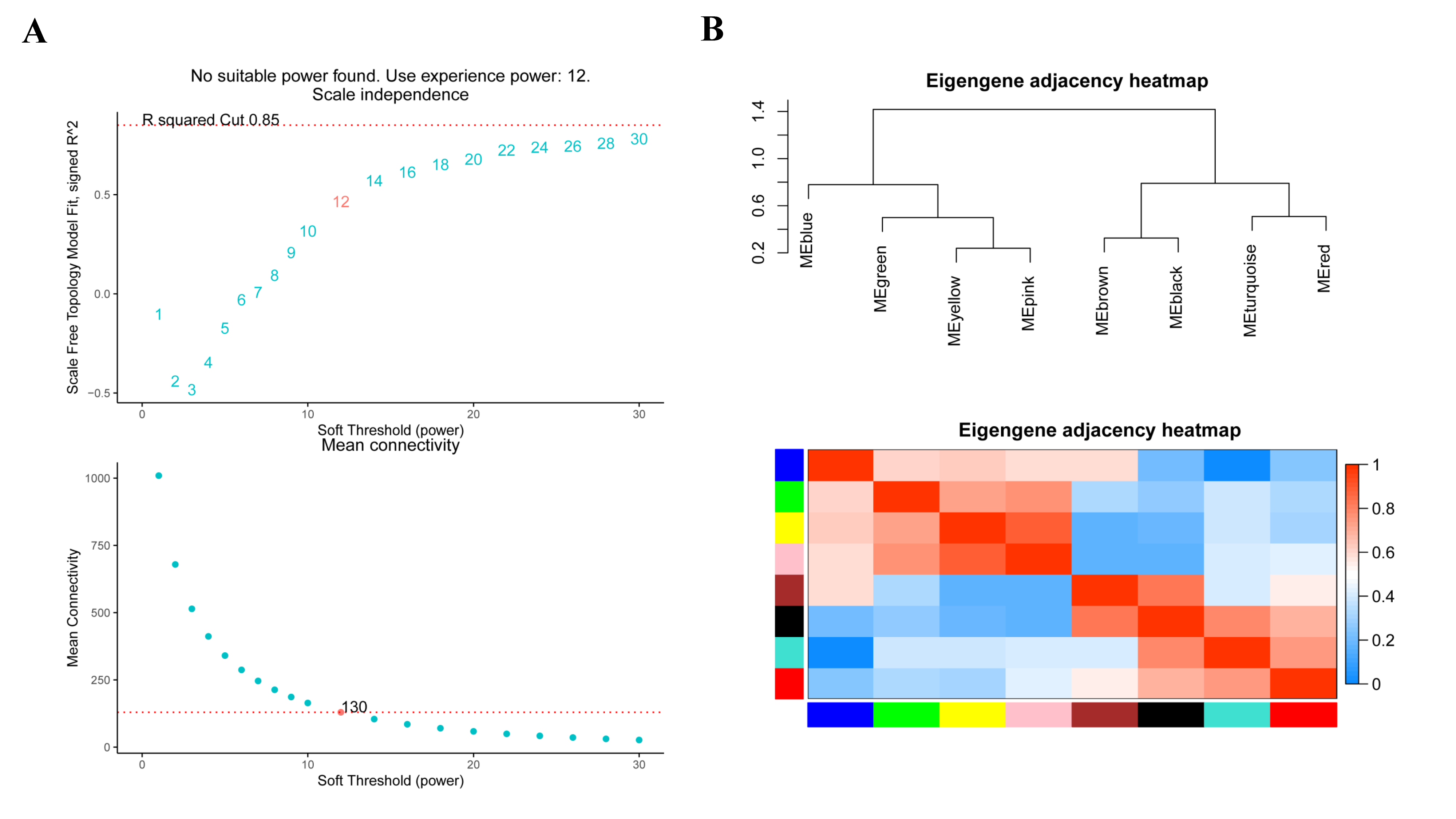

Supplement: Supplement Figure 4 — (A) soft-threshold power of GSE163154. (B) The heatmap of co-expression modules in GSE163154. [file Image4.tif]

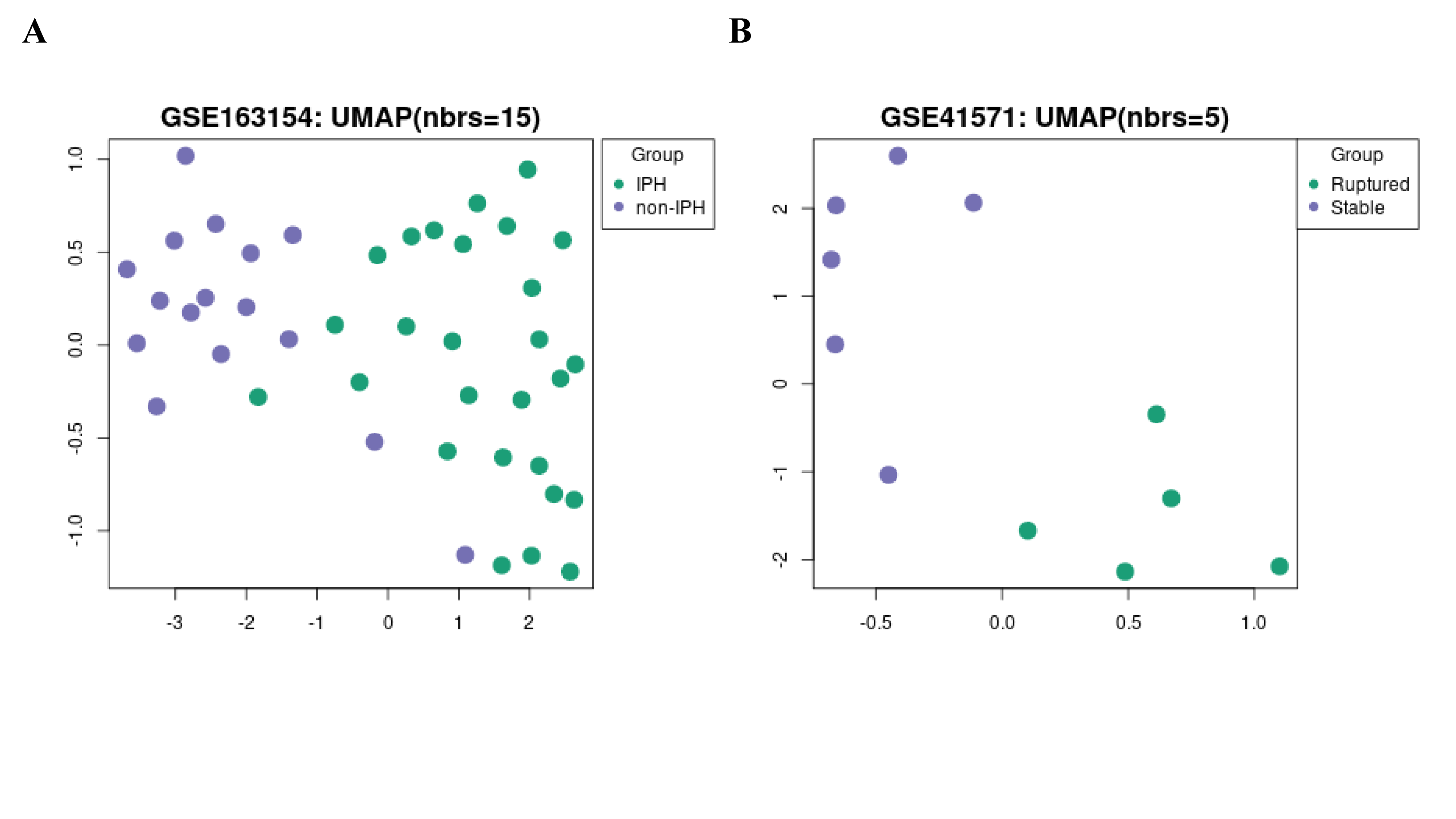

Supplement: Supplement Figure 5 — (A) The UMAP diagram of GSE163154. (B) The UMAP diagram of GSE41571. [file Image5.tif]

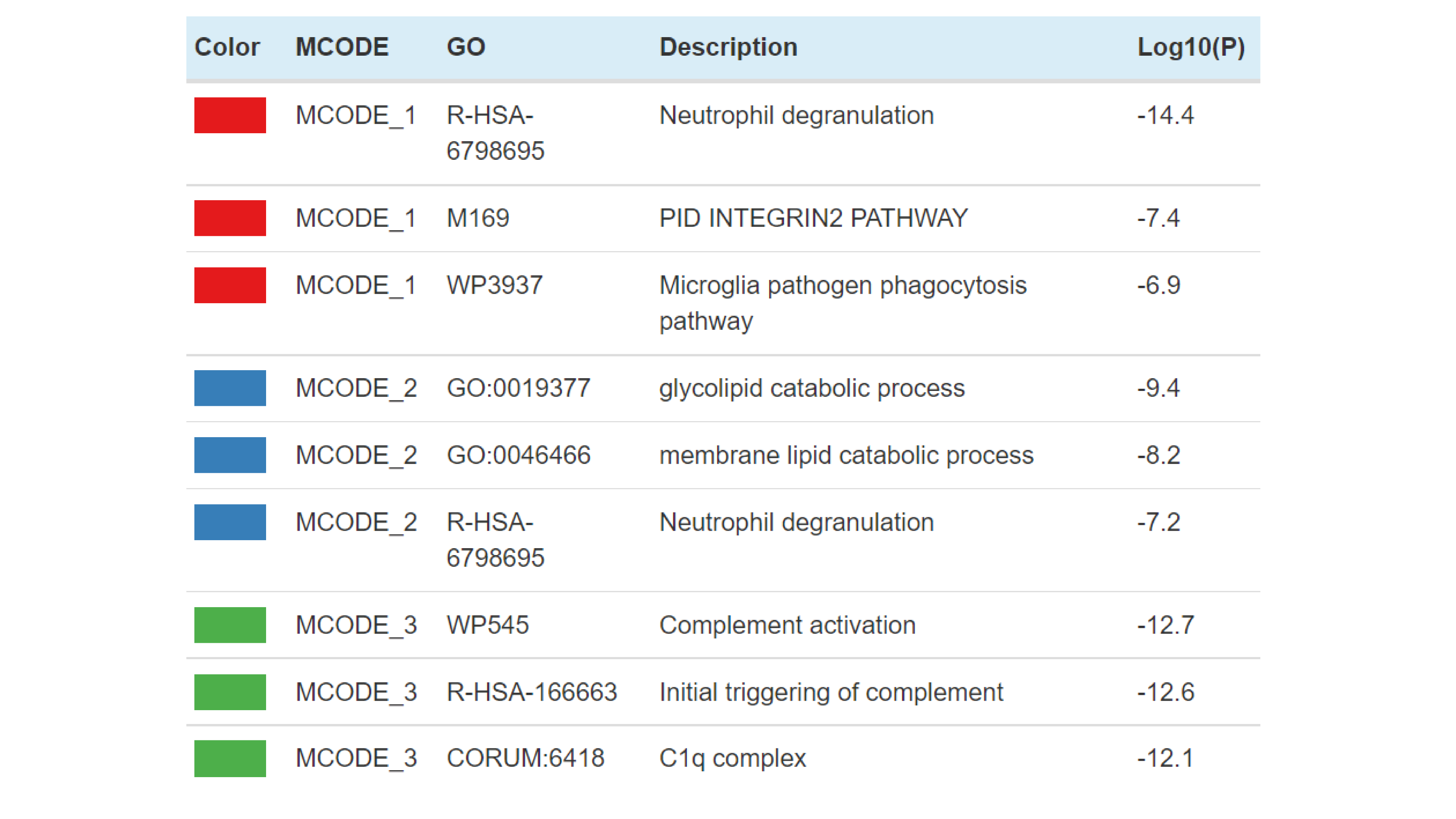

Supplement: Supplement Figure 6 — The enriched pathways of hub genes. [file Image6.tif]
